# Author Correction: Single-gold etching at the hypercarbon atom of *C*-centred hexagold(I) clusters protected by chiral *N*-heterocyclic carbenes

**DOI:** 10.1038/s41467-026-76884-8

**Published:** 2026-08-20

**Authors:** Xiao-Li Pei, Pei Zhao, Hitoshi Ube, Zhen Lei, Masahiro Ehara, Mitsuhiko Shionoya

**Affiliations:** 1 Department of Chemistry, Graduate School of Science, The University of Tokyo, Tokyo, 113-0033 Japan; 2 Research Centre for Computational Science, Institute for Molecular Science and SOKENDAI, Myodaiji, Okazaki, Aichi 444-8585 Japan; 3 Present Address: Research Institute for Science and Technology, Tokyo University of Science, 2641 Yamazaki, Noda, Chiba 278-8510 Japan; 4 Present Address: Fujian Provincial Key Laboratory of Advanced Inorganic Oxygenated Materials, College of Chemistry, Fuzhou University, Fuzhou, 350108 P. R. China

**Keywords:** Organometallic chemistry, Chemical synthesis, Nanoscale materials

Correction to: *Nature Communications* 10.1038/s41467-024-49295-w, published online 12 June 2024

In the version of this article initially published, in the first sentence of the Methods “Chemical reagents” section, there were two typographical errors where the current text “*cis*−1,2-bis(diphenylphosphino)ethene” and “1,2-bis(diphenylphosphino)benzene” originally read “*cis*−1,2-bis(diethylphosphino)ethene” and “2-bis(diethylphosphino)benzene”; the text is now amended in the HTML and PDF versions of the article.

