# Supplementary material for: Single-gold etching at the hypercarbon atom of C-centred hexagold(I) clusters protected by chiral N-heterocyclic carbenes
Source: Nat Commun. 2024 Jun 12;15:5024. doi: 10.1038/s41467-024-49295-w (PMC11169362; doi:10.1038/s41467-024-49295-w)
Supplement: Supplementary file 1 — Supplementary Information [file 41467_2024_49295_MOESM1_ESM.pdf]

Supplementary Information

Single-gold etching at the hypercarbon atom of *C*-centred hexagold(I) clusters protected by chiral *N*-heterocyclic carbenes

Xiao-Li Pei[1,3], Pei Zhao[2], Hitoshi Ube[1], Zhen Lei[1,4], Masahiro Ehara*[2] & Mitsuhiko Shionoya*[1,3]

[1]Department of Chemistry, Graduate School of Science, The University of Tokyo, Tokyo 113-0033, Japan

[2]Research Centre for Computational Science, Institute for Molecular Science and SOKENDAI, Myodaiji, Okazaki, Aichi 444-8585, Japan

[3]Current affiliation: Research Institute for Science and Technology, Tokyo University of Science, 2641 Yamazaki, Noda, Chiba 278-8510, Japan

[4]Current affiliation: Fujian Provincial Key Laboratory of Advanced Inorganic Oxygenated Materials, College of Chemistry, Fuzhou University, Fuzhou 350108, P. R. China

*Email: ehara@ims.ac.jp; shionoya@chem.s.u-tokyo.ac.jp

Table of Contents

Supplementary Figures 4

Supplementary Fig. 1. The schematic illustrations of the *C*-centred gold(I) clusters 4

Supplementary Fig. 2. ^{1}H NMR spectrum of *SS*-**2**$^{\mathbf{NHC}}$ in d_6-acetone (500 MHz, 300 K). 4

Supplementary Fig. 3. ^{13}C NMR spectrum of *SS*-**2**$^{\mathbf{NHC}}$ in d_6-acetone (126 MHz, 300 K). 5

Supplementary Fig. 4. ^{1}H–^{1}H COSY NMR spectrum of *SS*-**2**$^{\mathbf{NHC}}$ in d_6-acetone (300 K). 5

Supplementary Fig. 5. ^{13}C–^{1}H HSQC NMR spectrum of *SS*-**2**$^{\mathbf{NHC}}$ in d_6-acetone (300 K). 6

Supplementary Fig. 6. Comparison of ^{1}H NMR spectra of *SS*-**2**$^{\mathbf{NHC}}$ and *SS*-**1**$^{\mathbf{NHC}}$ in d_6-acetone (500 MHz, 300 K). 6

Supplementary Fig. 7. Comparison of ^{13}C NMR spectra of *SS*-**2**$^{\mathbf{NHC}}$ and *SS*-**1**$^{\mathbf{NHC}}$ in d_6-acetone (126 MHz, 300 K). 7

Supplementary Fig. 8. ^{31}P NMR spectrum of **2**$^{\mathbf{TPP}}$ in d_6-acetone (202 MHz, 300 K). 7

Supplementary Fig. 9. ^{1}H NMR spectrum of **2**$^{\mathbf{TPP}}$ in d_6-acetone (500 MHz, 300 K). 8

Supplementary Fig. 10. ^{13}C NMR spectrum of **2**$^{\mathbf{TPP}}$ in d_6-acetone (126 MHz, 300 K). 8

Supplementary Fig. 11. ^{1}H–^{1}H COSY NMR spectrum of **2**$^{\mathbf{TPP}}$ in d_6-acetone (300 K). 9

Supplementary Fig. 12. ^{13}C–^{1}H HSQC NMR spectrum of **2**$^{\mathbf{TPP}}$ in d_6-acetone (300 K). 9

Supplementary Fig. 13. Single-crystal X-ray structures of cationic parts in *SS*-**2**$^{\mathbf{NHC}}$, *RR*-**2**$^{\mathbf{NHC}}$ and **2**$^{\mathbf{TPP}}$ at the 50% thermal ellipsoid probability.. 10

Supplementary Fig. 14. Single-crystal X-ray structure of **3** at the 50% thermal ellipsoid probability. 10

Supplementary Fig. 15. Comparison of the single-crystal structures of *SS*-**2**$^{\mathbf{NHC}}$ (orange) and *SS*-**1**$^{\mathbf{NHC}}$ (grey). 10

Supplementary Fig. 16. The intermolecular interactions found in the packing structures of *SS*-**2**$^{\mathbf{NHC}}$. 11

Supplementary Fig. 17. UV-vis absorption spectra, plotted as the molar absorptivity of *SS*-**2**$^{\mathbf{NHC}}$, **2**$^{\mathbf{TPP}}$, *SS*-**1**$^{\mathbf{NHC}}$ and **1**$^{\mathbf{TPP}}$ in CH_2Cl_2 (293 K). 11

Supplementary Fig. 18. Excitation and emission spectra of *SS*-**2**$^{\mathbf{NHC}}$, **2**$^{\mathbf{TPP}}$, *SS*-**1**$^{\mathbf{NHC}}$ and **1**$^{\mathbf{TPP}}$ in the solid state (293 K). 12

Supplementary Fig. 19. Emission spectra of *SS*-**2**$^{\mathbf{NHC}}$ in acetone, inset: photo under light irradiation at 365 nm. 12

Supplementary Fig. 20. The absolute quantum yields (QYs) of *SS*-**2**$^{\mathbf{NHC}}$, **2**$^{\mathbf{TPP}}$, *SS*-**1**$^{\mathbf{NHC}}$ and **1**$^{\mathbf{TPP}}$ in the solid state. 13

Supplementary Fig. 21. Lifetimes of *SS*-**2**$^{\mathbf{NHC}}$, **2**$^{\mathbf{TPP}}$, *SS*-**1**$^{\mathbf{NHC}}$ and **1**$^{\mathbf{TPP}}$ in the solid state. 13

Supplementary Fig. 22. TD-DFT calculated UV-vis absorption spectrum of *SS*-**2**$^{\mathbf{NHC}}$ in CH_2Cl_2. 14

Supplementary Fig. 23. TD-DFT calculated UV-vis absorption spectrum of **2**$^{\mathbf{TPP}}$ in CH_2Cl_2. 14

Supplementary Fig. 24. TD-DFT calculated UV-vis absorption spectrum of **1**$^{\mathbf{TPP}}$ in CH_2Cl_2. 14

Supplementary Fig. 25. The frontier molecular orbitals in *SS*-**2**$^{\mathbf{NHC}}$ calculated by DFT. 15

Supplementary Fig. 26. The frontier molecular orbitals in **2**$^{\mathbf{TPP}}$ calculated by DFT. 16

Supplementary Fig. 27. The frontier molecular orbitals in **1**$^{\mathbf{TPP}}$ calculated by DFT. 17

Supplementary Fig. 28. Time-course UV-vis absorption spectra of the etching reaction of **1**$^{\mathbf{TPP}}$ ($c = 5 \times 10^{-5}$ M, 293 K) using *cis*-depe in dichloromethane. 18

Supplementary Fig. 29. Time-course ^{1}H NMR spectra of *SS*-**1**$^{\mathbf{NHC}}$ etched with *cis*-depe (d_6-acetone, 300 K)...18
Supplementary Fig. 30. Time-course ^{1}H NMR spectra of **1**$^{\mathbf{TPP}}$ etched with *cis*-depe (d_6-acetone, 300 K)........19
Supplementary Fig. 31. The ESI-MS spectra of monitoring the reaction of *SS*-**1**$^{\mathbf{NHC}}$ etched with *cis*-depe. 20
Supplementary Fig. 32. The ESI-MS spectra of monitoring the reaction of **1**$^{\mathbf{TPP}}$ and *cis*-depe. 20
Supplementary Fig. 33. Calculated energy profiles for the proposed etching mechanism.................................21
Supplementary Fig. 34. ^{1}H NMR spectra showing stability studies of *SS*-**2**$^{\mathbf{NHC}}$ in d_6-acetone for one week. 22
Supplementary Fig. 35. ^{1}H NMR spectrum of *SS*-**2**$^{\mathbf{NHC}}$ in $CDCl_3$ (500 M, 300 K)...22
Supplementary Fig. 36. ^{31}P NMR and ^{1}H NMR spectra of crystalline **2**$^{\mathbf{TPP}}$ in $CDCl_3$ (300 K)..........................23
Supplementary Fig. 37. Single-crystal X-ray structures of the Cl-coordinated **CAu$^{\mathbf{I}}_{\mathbf{6}}$** clusters. 24
Supplementary Fig. 38. ^{1}H NMR spectrum of *SS*-**4**$^{\mathbf{NHC}}$ in d_6-acetone (500 MHz, 300 K). 25
Supplementary Fig. 39. ^{13}C NMR spectrum of *SS*-**4**$^{\mathbf{NHC}}$ in d_6-acetone (126 MHz, 300 K). 25
Supplementary Fig. 40. ^{1}H–^{1}H COSY NMR spectrum of *SS*-**4**$^{\mathbf{NHC}}$ in d_6-acetone (300 K). 26
Supplementary Fig. 41. ^{13}C–^{1}H HSQC NMR spectrum of *SS*-**4**$^{\mathbf{NHC}}$ in d_6-acetone (300 K). 26
Supplementary Fig. 42. CD spectra of the *C*-centred gold(I) clusters. 27
Supplementary Tables...28
Supplementary Table 1. Crystallographic data of *SS*-**2**$^{\mathbf{NHC}}$, *RR*-**2**$^{\mathbf{NHC}}$ and **2**$^{\mathbf{TPP}}$. 28
Supplementary Table 2. Important bond lengths and angles of *SS*-**2**$^{\mathbf{NHC}}$, *RR*-**2**$^{\mathbf{NHC}}$ and **2**$^{\mathbf{TPP}}$. 29
Supplementary Table 3. Bond length (*d*, Å) and Wiberg bond order (WBO) of C_{centre}–Au^I and $Au^I \cdots Au^I$ in different CAu^I_n (n = 5, 6) clusters. 29
Supplementary Table 4. Excited states of *SS*-**2**$^{\mathbf{NHC}}$ with oscillator strength (*f*) larger than 0.04 calculated by TD-DFT. 30
Supplementary Table 5. Excited states of **2**$^{\mathbf{TPP}}$ with oscillator strength (*f*) larger than 0.04 calculated by TD-DFT. 31
Supplementary Table 6. Orbital composition analysis with Mulliken partition of *SS*-**2**$^{\mathbf{NHC}}$. 32
Supplementary Table 7. Orbital composition analysis with Mulliken partition of **2**$^{\mathbf{TPP}}$......................................32
Supplementary Table 8. Orbital composition analysis with Mulliken partition of **1**$^{\mathbf{TPP}}$......................................32
Supplementary Table 9. TD-DFT calculated emission values of *SS*-**1**$^{\mathbf{NHC}}$ and *SS*-**2**$^{\mathbf{NHC}}$. 32
Supplementary Table 10. Crystallographic data of *SS*-**4**$^{\mathbf{NHC}}$ and *RR*-**4**$^{\mathbf{NHC}}$...33
Supplementary Table 11. Important bond lengths and angles of *SS*-**4**$^{\mathbf{NHC}}$ and *RR*-**4**$^{\mathbf{NHC}}$. 34
Supplementary Note 34
Supplementary References...34

Supplementary Figures

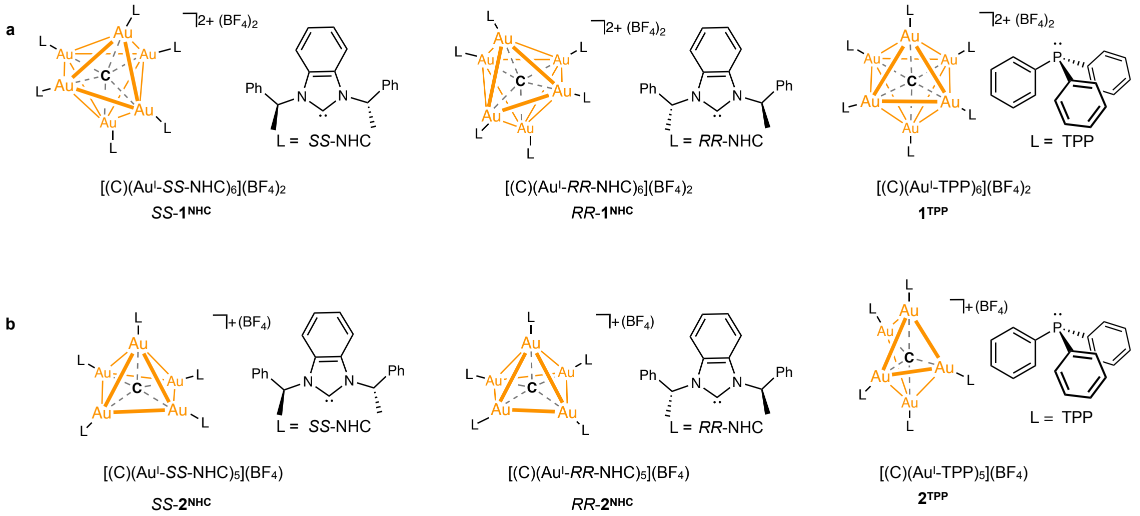

Supplementary Fig. 1. The schematic illustrations of the *C*-centred gold(I) clusters.
a The $\mathbf{CAu^{I}_{6}}$ clusters: [(C)(Au^I-*SS*-NHC)$_6$](BF_4)$_2$ (*SS*-$\mathbf{1}^{\mathbf{NHC}}$)[1], [(C)($Au^I$-*RR*-NHC)$_6$]($BF_4$)$_2$ (*RR*-$\mathbf{1}^{\mathbf{NHC}}$)[9], [(C)($Au^I$-TPP)$_6$]($BF_4$)$_2$ ($\mathbf{1}^{\mathbf{TPP}}$)[2]. **b** The $\mathbf{CAu^{I}_{5}}$ clusters: [(C)(Au^I-*SS*-NHC)$_5$](BF_4) (*SS*-$\mathbf{2}^{\mathbf{NHC}}$), [(C)($Au^I$-*RR*-NHC)$_5$]($BF_4$) (*RR*-$\mathbf{2}^{\mathbf{NHC}}$), [(C)($Au^I$-TPP)$_5$]($BF_4$) ($\mathbf{2}^{\mathbf{TPP}}$)[3].
Note: another $Au^I\cdots Au^I$ interaction was observed in the structure of $\mathbf{2}^{\mathbf{TPP}}$, which was slightly different from the reported data[14].

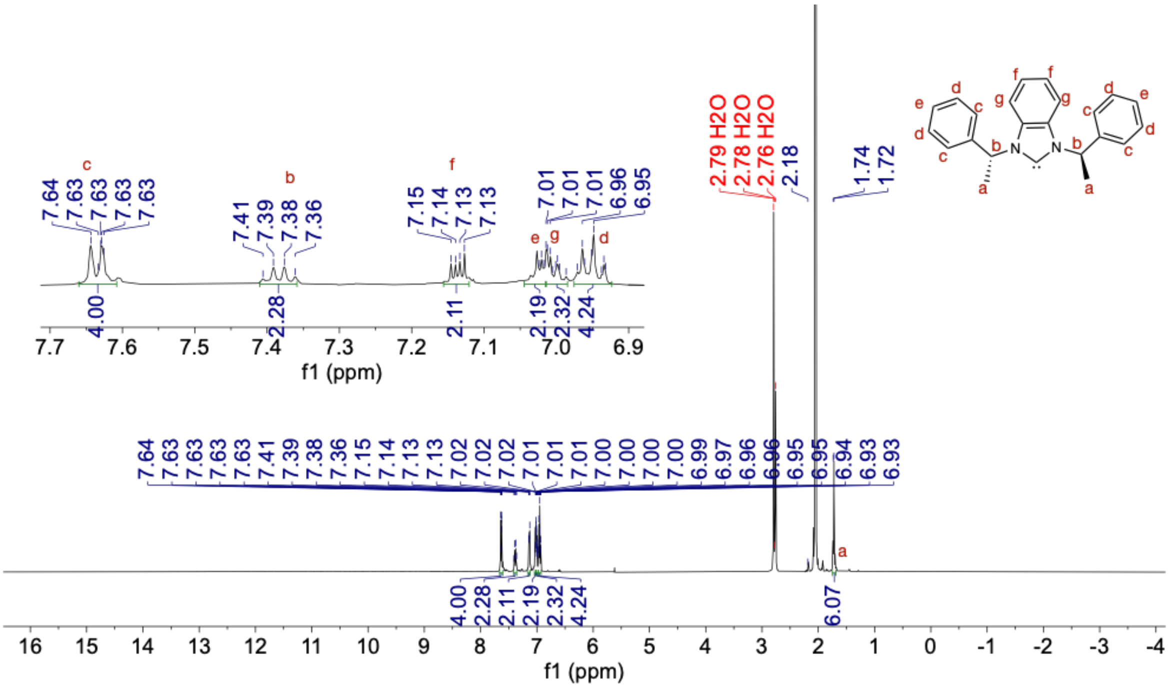

Supplementary Fig. 2. ^{1}H NMR spectrum of *SS*-$\mathbf{2}^{\mathbf{NHC}}$ in d_6-acetone (500 MHz, 300 K).

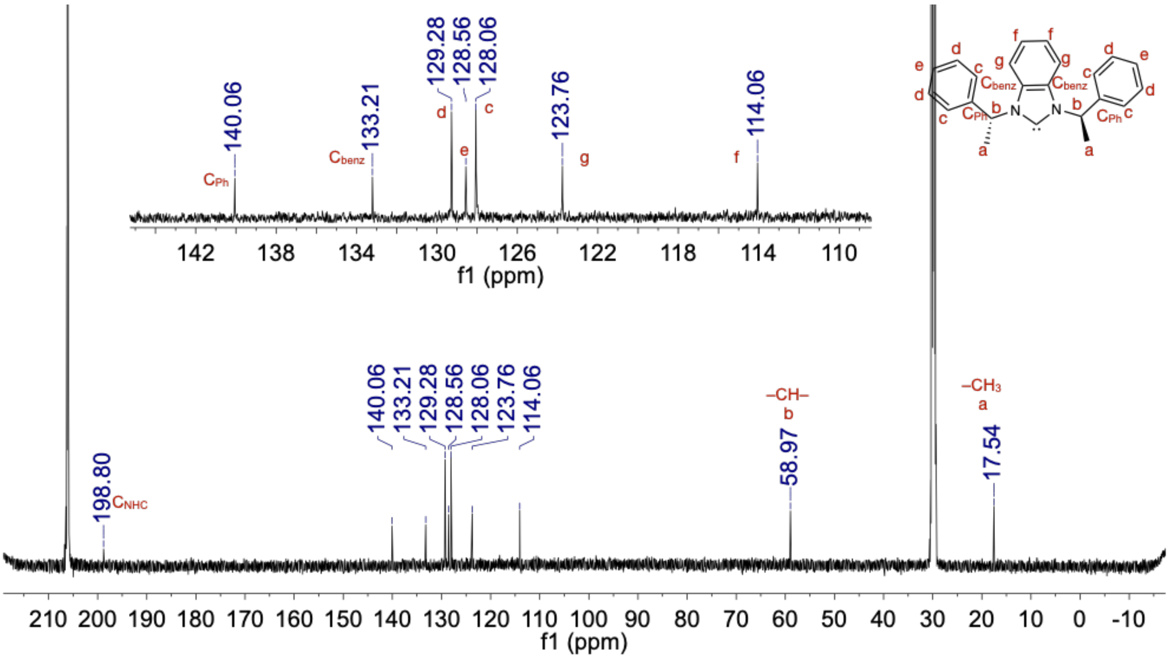

Supplementary Fig. 3. ^{13}C NMR spectrum of *SS*-**2**$^{\mathbf{NHC}}$ in d_6-acetone (126 MHz, 300 K).

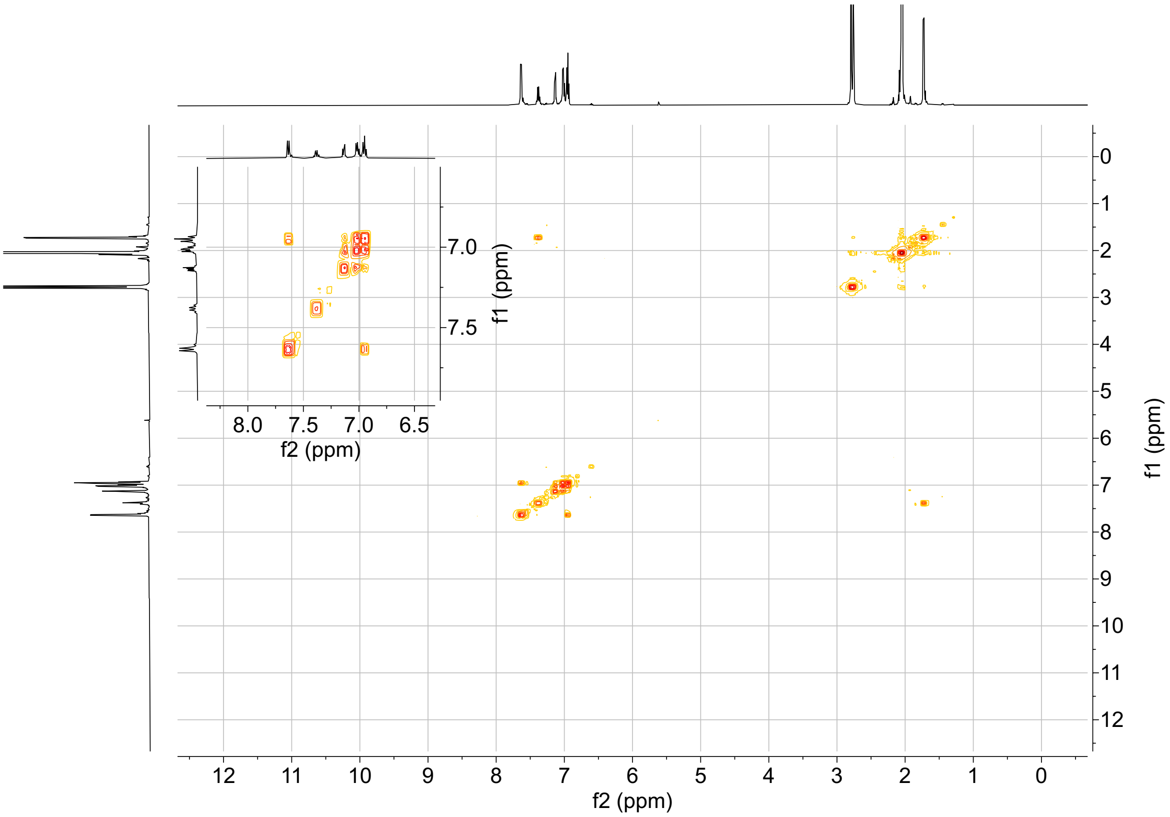

Supplementary Fig. 4. ^{1}H–^{1}H COSY NMR spectrum of *SS*-**2**$^{\mathbf{NHC}}$ in d_6-acetone (300 K).

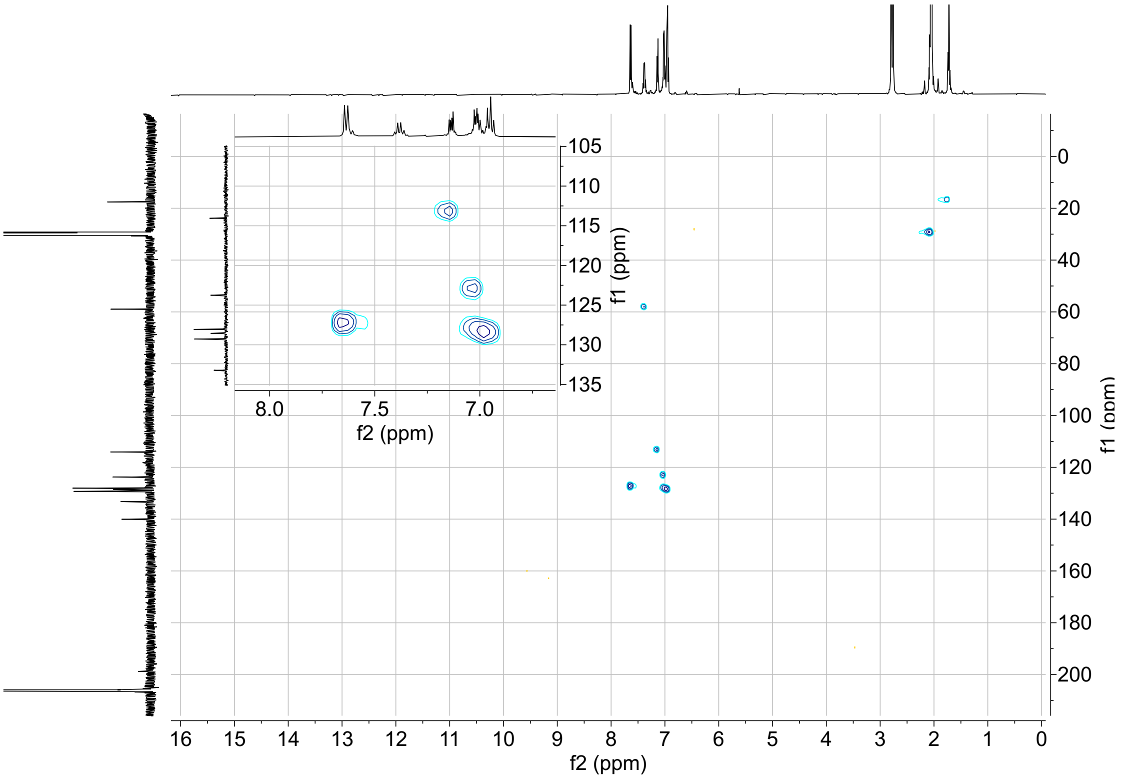

Supplementary Fig. 5. ^{13}C–^{1}H HSQC NMR spectrum of *SS*-**2**^{NHC} in d_6-acetone (300 K).

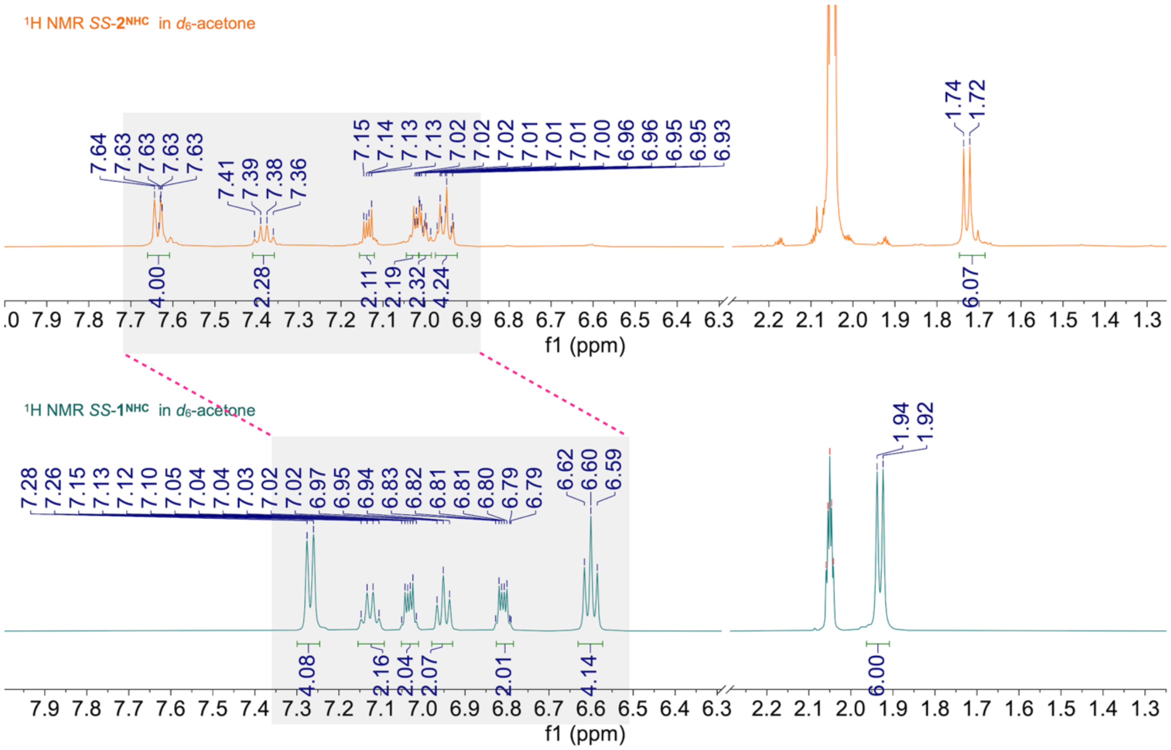

Supplementary Fig. 6. Comparison of ^{1}H NMR spectra of *SS*-**2**^{NHC} and *SS*-**1**^{NHC} in d_6-acetone (500 MHz, 300 K).

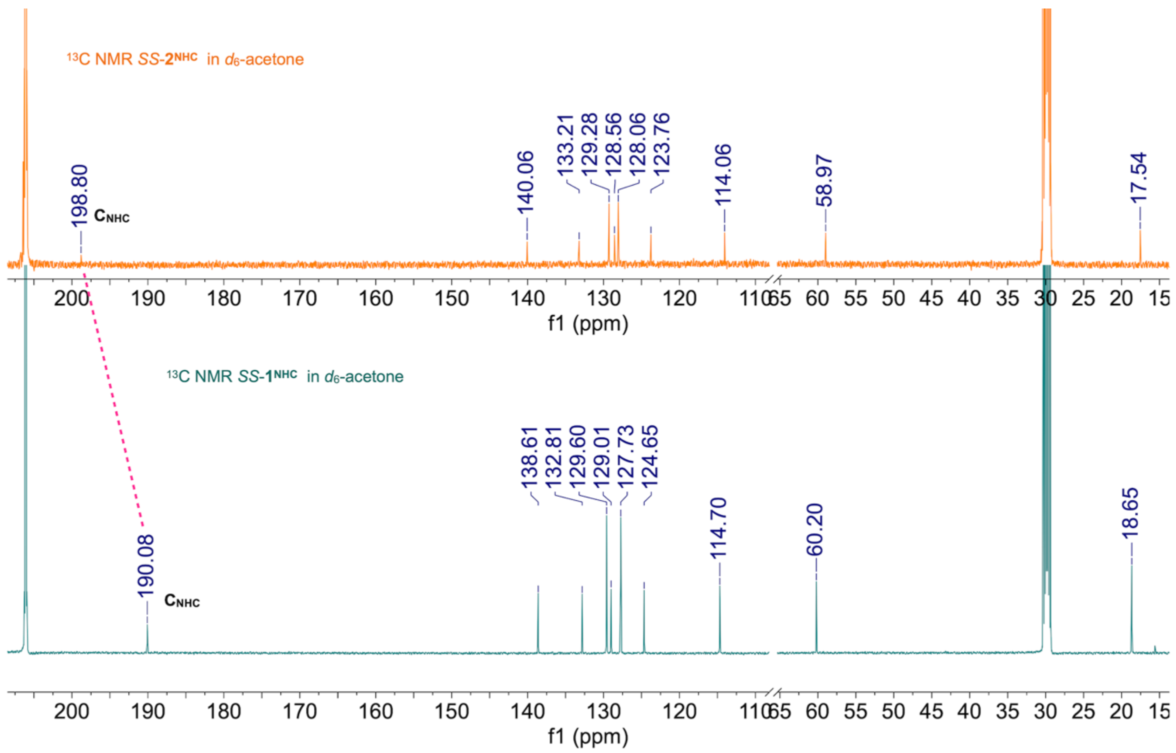

Supplementary Fig. 7. Comparison of ^{13}C NMR spectra of *SS*-**2**$^{\mathbf{NHC}}$ and *SS*-**1**$^{\mathbf{NHC}}$ in d_6-acetone (126 MHz, 300 K).

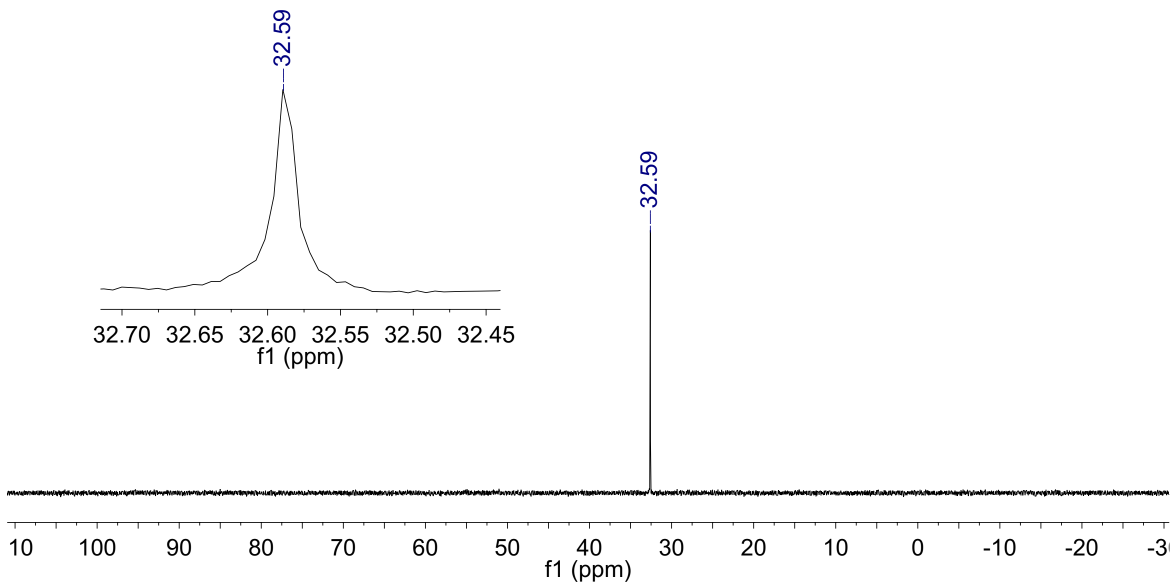

Supplementary Fig. 8. ^{31}P NMR spectrum of **2**$^{\mathbf{TPP}}$ in d_6-acetone (202 MHz, 300 K).

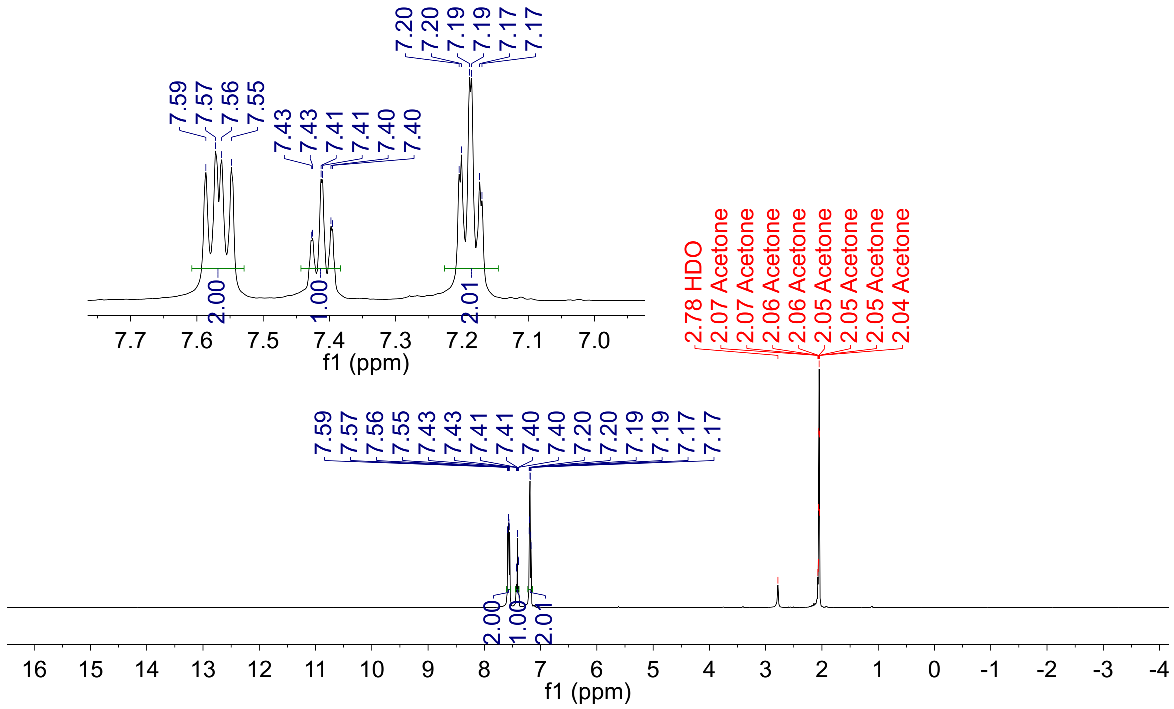

Supplementary Fig. 9. ^{1}H NMR spectrum of $\mathbf{2^{TPP}}$ in d_6-acetone (500 MHz, 300 K).

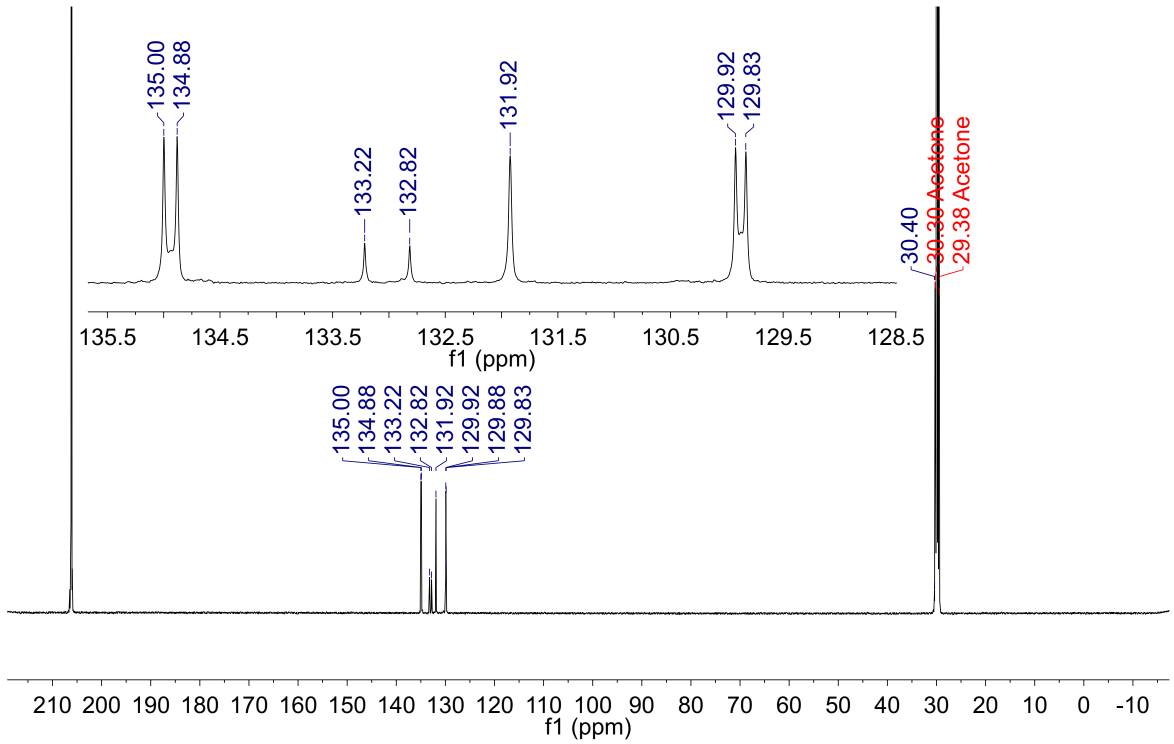

Supplementary Fig. 10. ^{13}C NMR spectrum of $\mathbf{2^{TPP}}$ in d_6-acetone (126 MHz, 300 K).

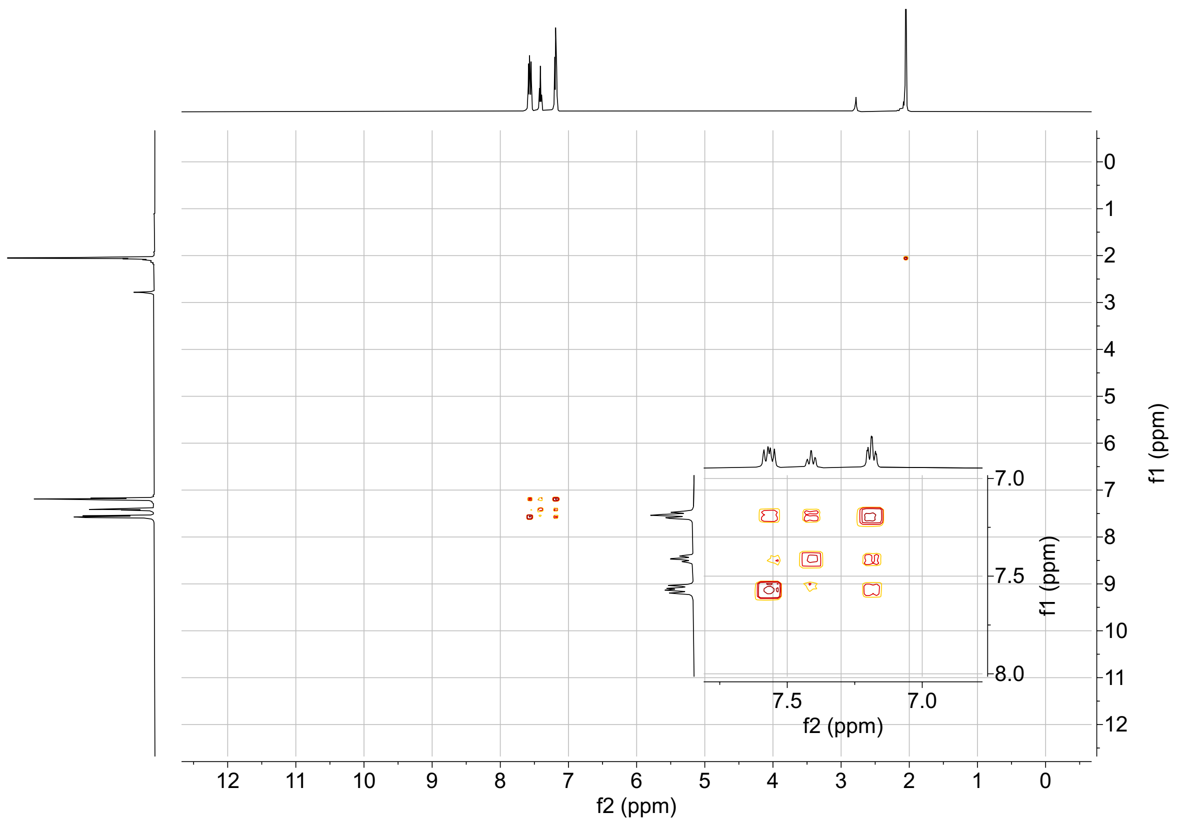

Supplementary Fig. 11. ^{1}H–^{1}H COSY NMR spectrum of **2**$^{\mathbf{TPP}}$ in d_6-acetone (300 K).

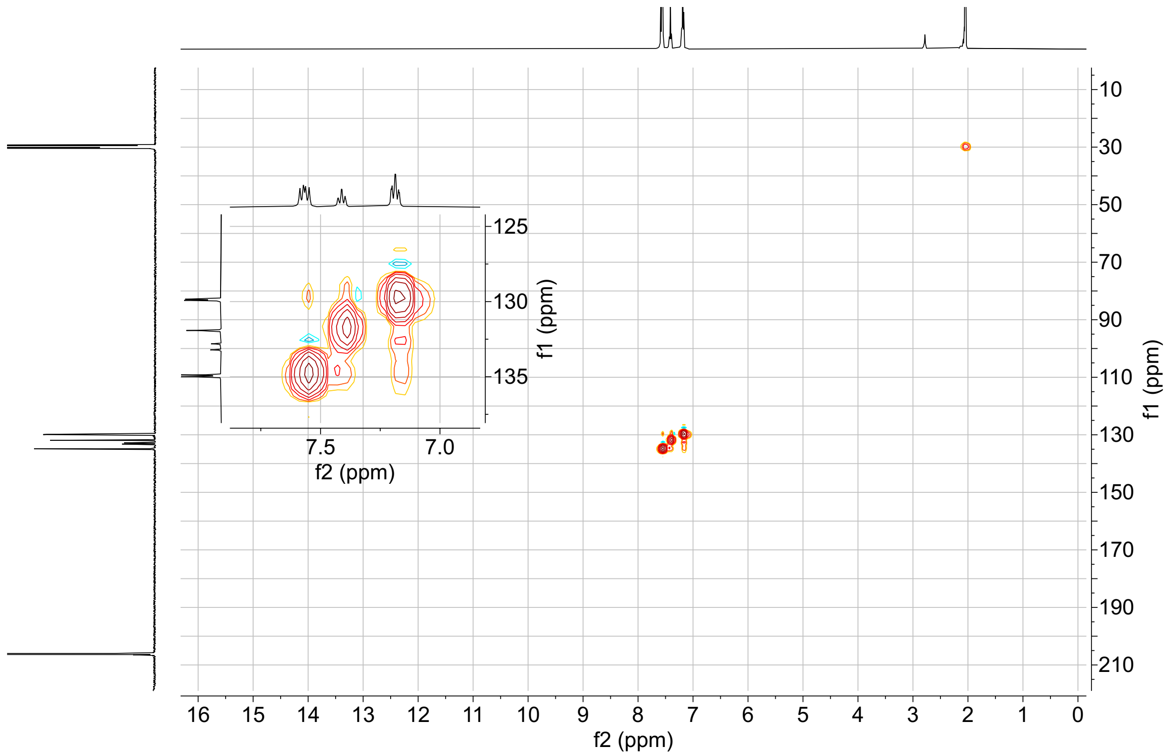

Supplementary Fig. 12. ^{13}C–^{1}H HSQC NMR spectrum of **2**$^{\mathbf{TPP}}$ in d_6-acetone (300 K).

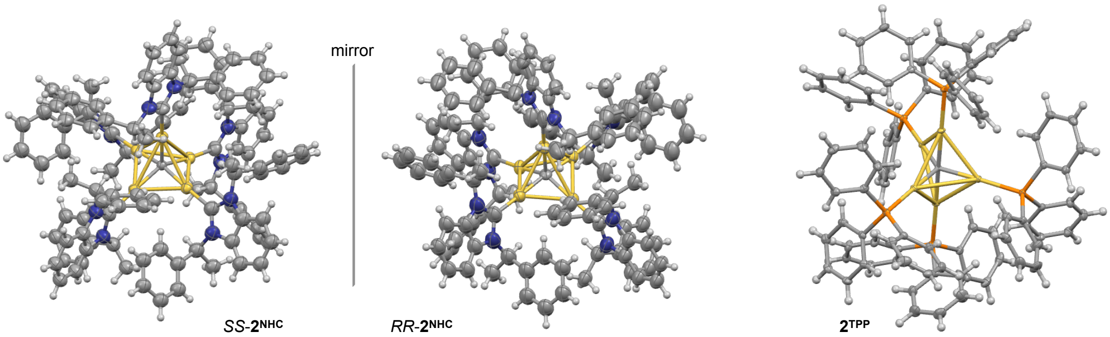

Supplementary Fig. 13. Single-crystal X-ray structures of cationic parts in *SS*-**2**$^{\mathbf{NHC}}$, *RR*-**2**$^{\mathbf{NHC}}$ and **2**$^{\mathbf{TPP}}$ at the 50% thermal ellipsoid probability. Colour code: Au, yellow; C, grey; N, blue; P, orange; H, white. BF_4^- counterions and solvent molecules are omitted for clarity.

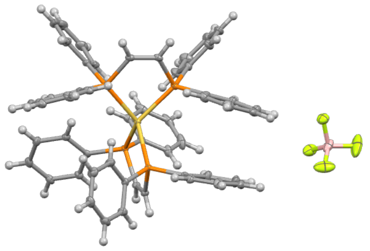

Supplementary Fig. 14. Single-crystal X-ray structure of **3** at the 50% thermal ellipsoid probability. Colour code: Au, yellow; C, grey; N, blue; B, pink; F, lime; P, orange; H, white. This SCXRD data is consistent with the reported data[4].

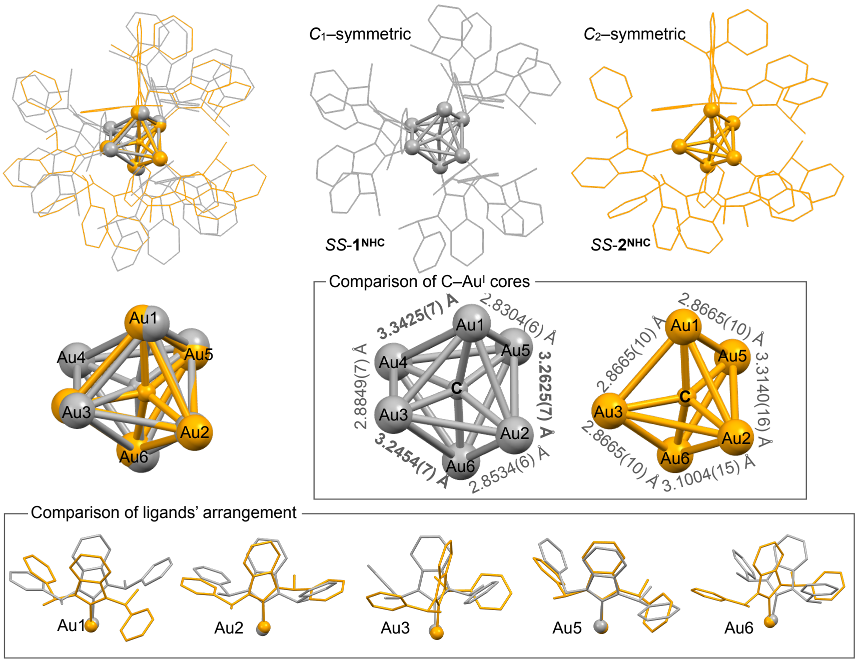

Supplementary Fig. 15. Comparison of the single-crystal structures of *SS*-**2**$^{\mathbf{NHC}}$ (orange) and *SS*-**1**$^{\mathbf{NHC}}$ (grey).

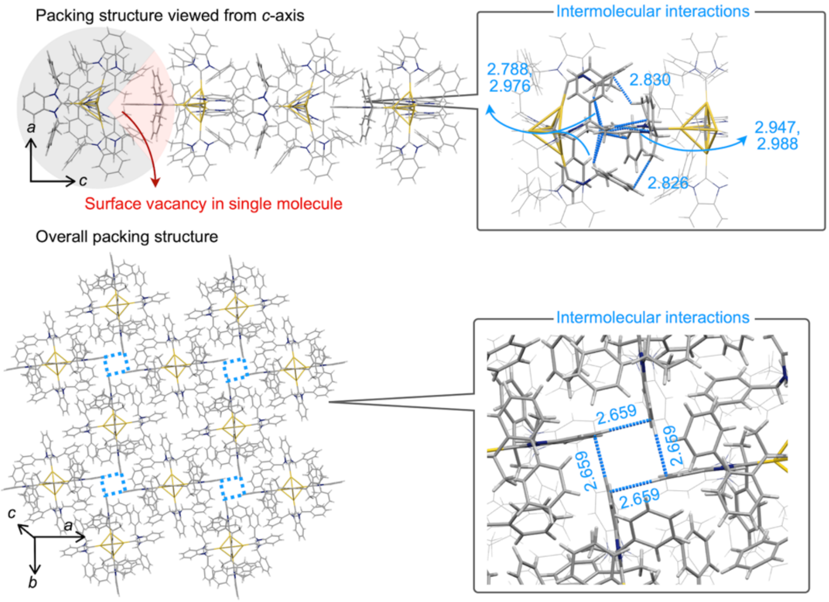

Supplementary Fig. 16. The intermolecular interactions found in the packing structures of *SS*-$\mathbf{2}^{\mathbf{NHC}}$.

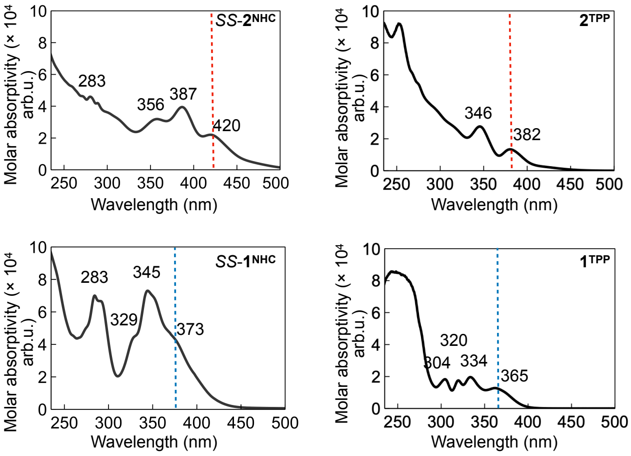

Supplementary Fig. 17. UV-vis absorption spectra, plotted as the molar absorptivity of *SS*-$\mathbf{2}^{\mathbf{NHC}}$, $\mathbf{2}^{\mathbf{TPP}}$, *SS*-$\mathbf{1}^{\mathbf{NHC}}$ and $\mathbf{1}^{\mathbf{TPP}}$ in CH_2Cl_2 (293 K).

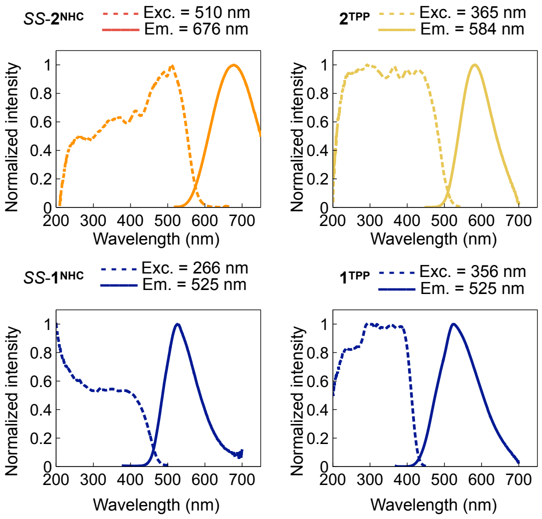

Supplementary Fig. 18. Excitation and emission spectra of *SS*-**2**$^{\mathbf{NHC}}$, **2**$^{\mathbf{TPP}}$, *SS*-**1**$^{\mathbf{NHC}}$ and **1**$^{\mathbf{TPP}}$ in the solid state (293 K).

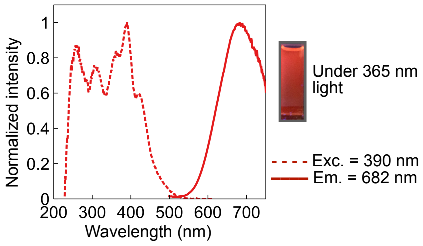

Supplementary Fig. 19. Emission spectra of *SS*-**2**$^{\mathbf{NHC}}$ in acetone, inset: photo under light irradiation at 365 nm. Once the sample of *SS*-**2**$^{\mathbf{NHC}}$ was dissolved, the spectrum was measured immediately (293 K).

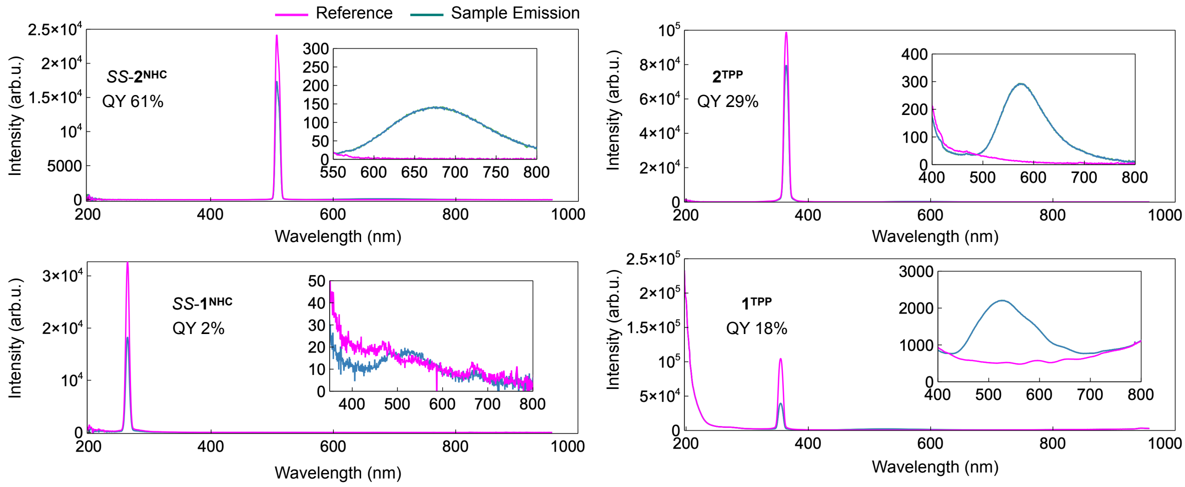

Supplementary Fig. 20. The absolute quantum yields (QYs) of *SS*-$\mathbf{2}^{\mathrm{NHC}}$, $\mathbf{2}^{\mathrm{TPP}}$, *SS*-$\mathbf{1}^{\mathrm{NHC}}$ and $\mathbf{1}^{\mathrm{TPP}}$ in the solid state.

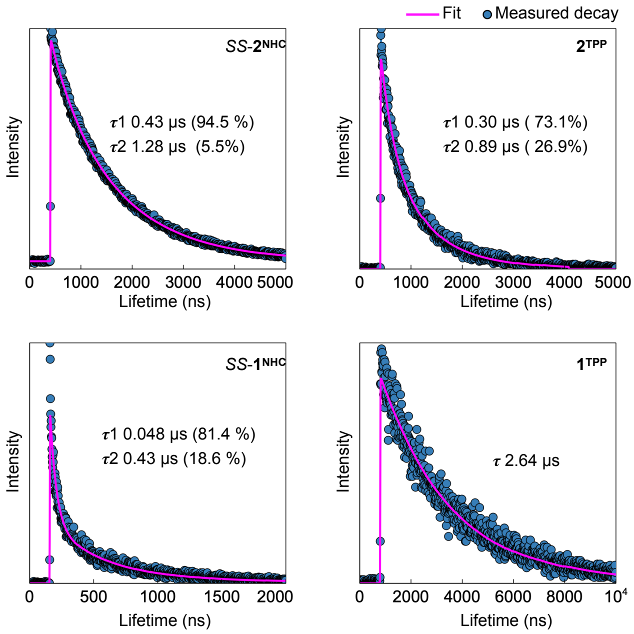

Supplementary Fig. 21. Lifetimes of *SS*-$\mathbf{2}^{\mathrm{NHC}}$, $\mathbf{2}^{\mathrm{TPP}}$, *SS*-$\mathbf{1}^{\mathrm{NHC}}$ and $\mathbf{1}^{\mathrm{TPP}}$ in the solid state.

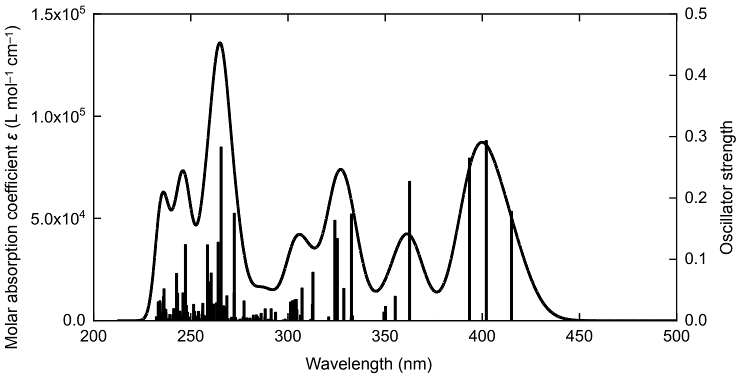

Supplementary Fig. 22. TD-DFT calculated UV-vis absorption spectrum of *SS*-**2**^NHC^ in CH_2Cl_2.

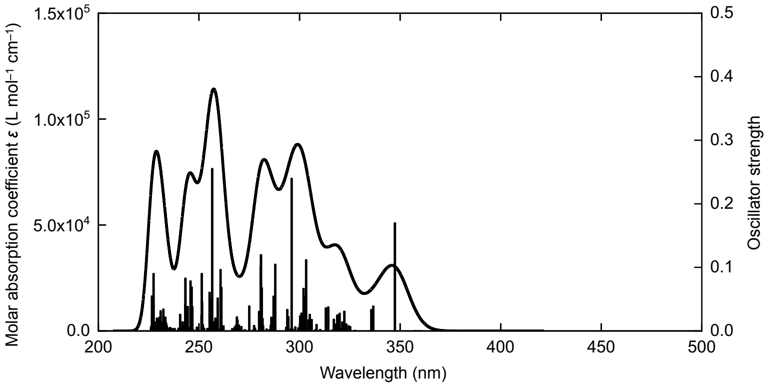

Supplementary Fig. 23. TD-DFT calculated UV-vis absorption spectrum of **2**^TPP^ in CH_2Cl_2.

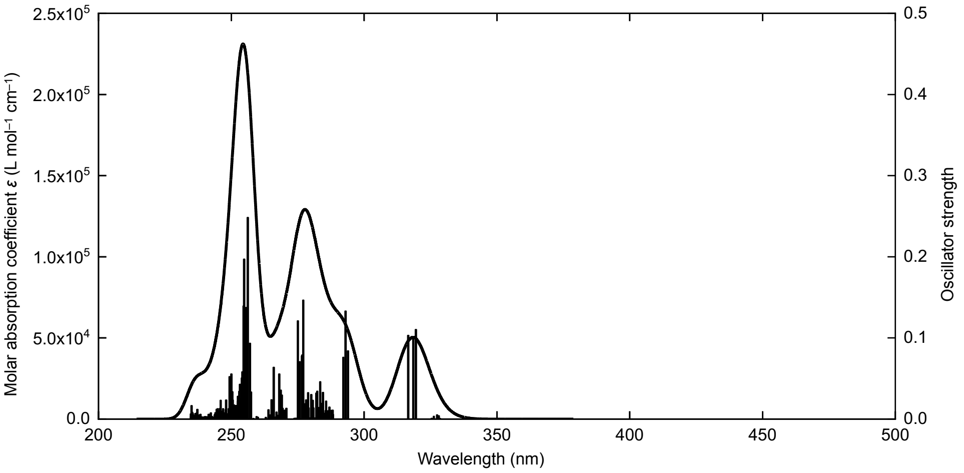

Supplementary Fig. 24. TD-DFT calculated UV-vis absorption spectrum of **1**^TPP^ in CH_2Cl_2.

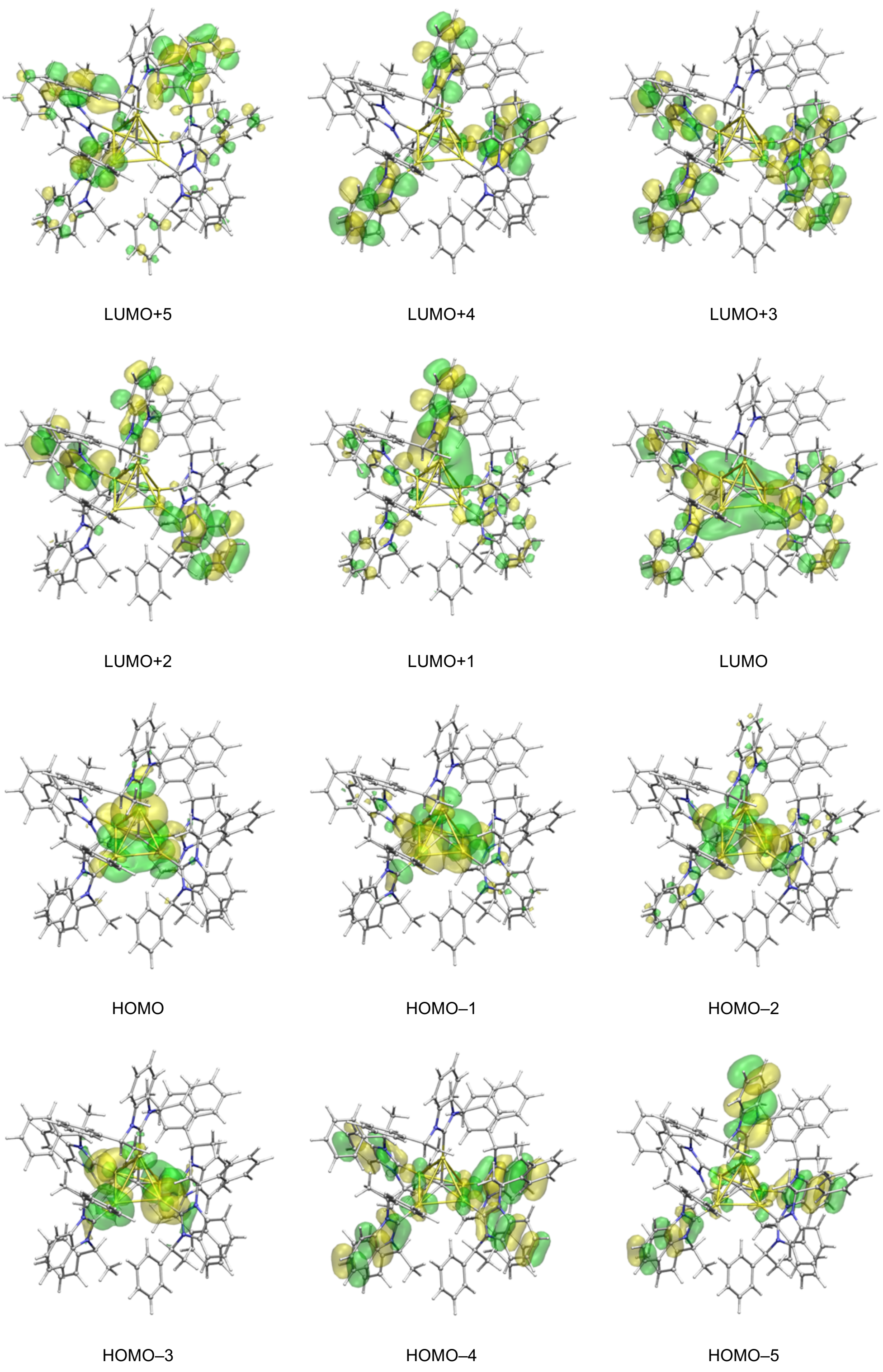

Supplementary Fig. 25. The frontier molecular orbitals in *SS*-**2**$^{\mathbf{NHC}}$ calculated by DFT.
Yellow and green represent different phases of molecular orbitals. Colour code for atoms: Au, yellow; C, grey; N, blue; H, white.

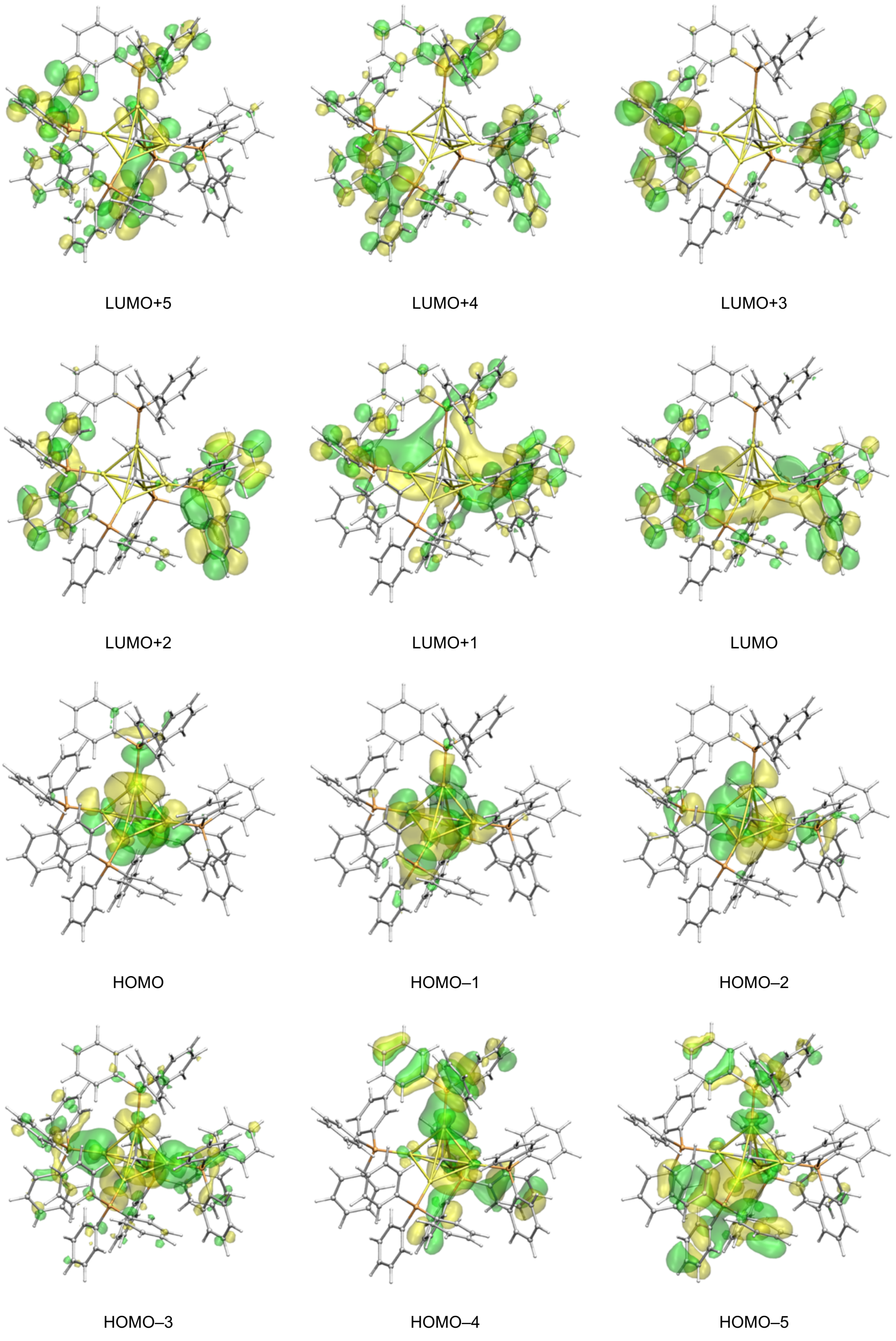

Supplementary Fig. 26. The frontier molecular orbitals in **2**$^{\mathbf{TPP}}$ calculated by DFT.
Yellow and green represent different phases of molecular orbitals. Colour code for atoms: Au, yellow; C, grey; P, orange; H, white.

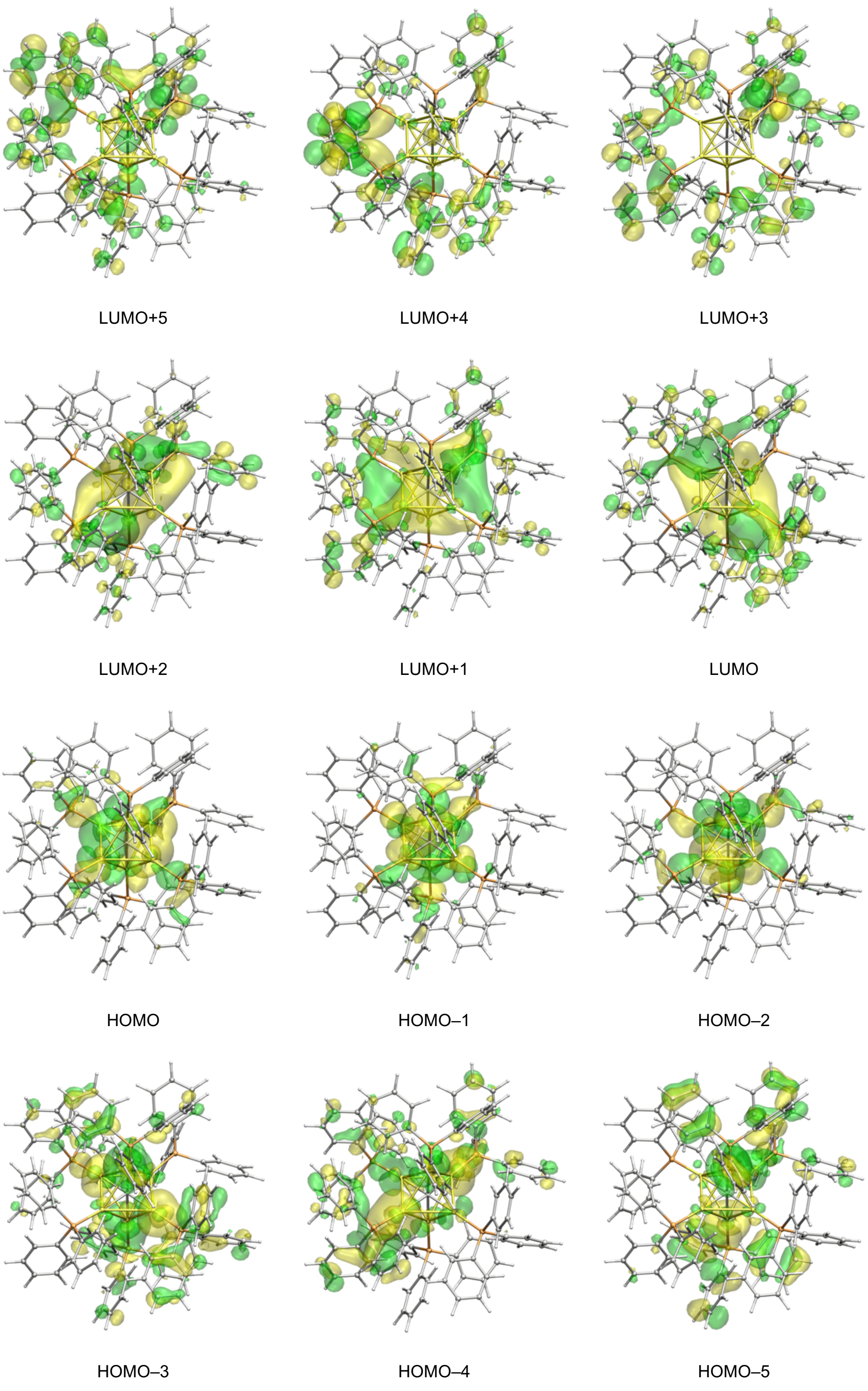

Supplementary Fig. 27. The frontier molecular orbitals in $\mathbf{1}^{\mathrm{TPP}}$ calculated by DFT.

Yellow and green represent different phases of molecular orbitals. Colour code for atoms: Au, yellow; C, grey; P, orange; H, white.

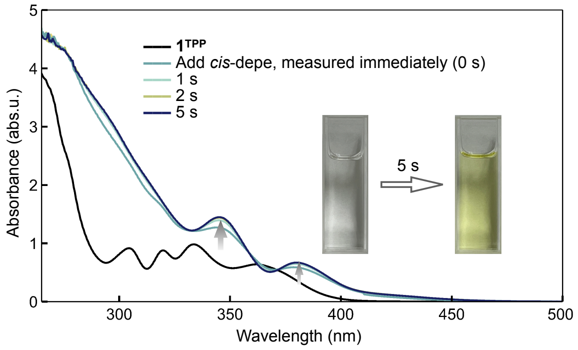

Supplementary Fig. 28. Time-course UV-vis absorption spectra of the etching reaction of $\mathbf{1}^{\mathrm{TPP}}$ (c = 5 × 10^{-5} M, 293 K) using *cis*-depe in dichloromethane. Insets: photographs: photographs of the reaction solution under ambient light irradiation at the beginning and after 5 s.

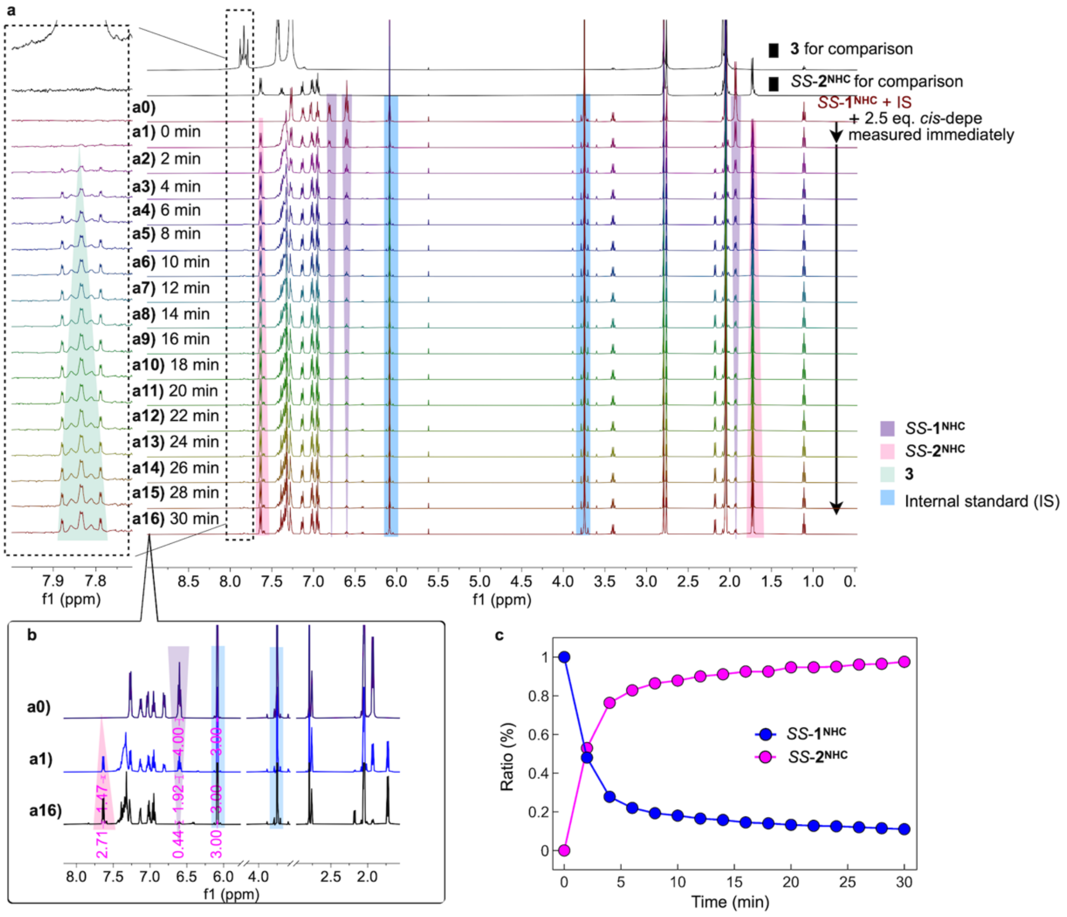

Supplementary Fig. 29. Time-course ^{1}H NMR spectra of *SS*-$\mathbf{1}^{\mathrm{NHC}}$ etched with *cis*-depe (d_6-acetone, 300 K). **a** Time-course ^{1}H NMR spectra: a0) *SS*-$\mathbf{1}^{\mathrm{NHC}}$ (3.3 mg, 1 μmol) with internal standard (IS, 1,3,5-trimethoxybenzene, 6 equiv., 6 μmol), a1) adding the solution of *cis*-depe (2.5 equiv., 3.3 × 10^{-2} M, 75 μL) in d_6-acetone into a0, and measured immediately, a2–a16) ^{1}H NMR spectra of this reaction recorded in intervals of 2 min. **b** ^{1}H NMR spectra of a0, a1, and a16 with integration. **c** The ratio of remaining

SS-**1**$^{\mathbf{NHC}}$ (blue) and the yield of *SS*-**2**$^{\mathbf{NHC}}$ (magenta) according to the spectra in a.

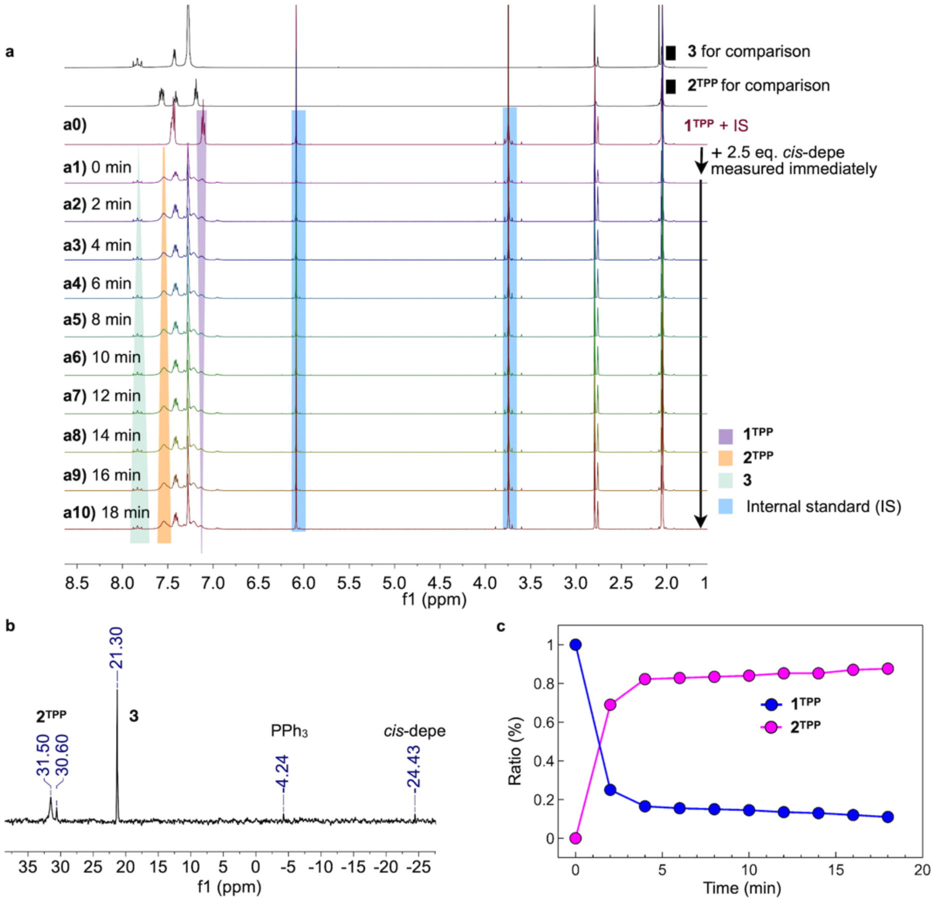

Supplementary Fig. 30. Time-course ^{1}H NMR spectra of **1**$^{\mathbf{TPP}}$ etched with *cis*-depe (d_6-acetone, 300 K). **a** Time-course ^{1}H NMR spectra: a0) **1**$^{\mathbf{TPP}}$ (2.9 mg, 1 μmol) with internal standard (IS, 1,3,5-trimethoxybenzene, 6 equiv., 6 μmol), a1) adding the solution of *cis*-depe (2.5 equiv., 3.3 × 10^{-2} M, 75 μL) in d_6-acetone into a0, and measured immediately, a2–a10) ^{1}H NMR spectra of this reaction recorded at intervals of 2 min. **b** ^{31}P NMR spectrum (d_6-acetone, 300 K) after 18 min, slightly shifted signals in this mixed reaction compared with the corresponding pure compounds: **2**$^{\mathbf{TPP}}$ (observed δ 31.5 ppm, pure δ 32.6 ppm), **3** (observed δ 21.3 ppm, pure δ 22.4 ppm), PPh_3 (observed δ –4.2 ppm, pure δ –5.5 ppm). **c** The ratio of remaining **1**$^{\mathbf{TPP}}$ (blue) and the yield of **2**$^{\mathbf{TPP}}$ (magenta) according to the spectra in **a**.

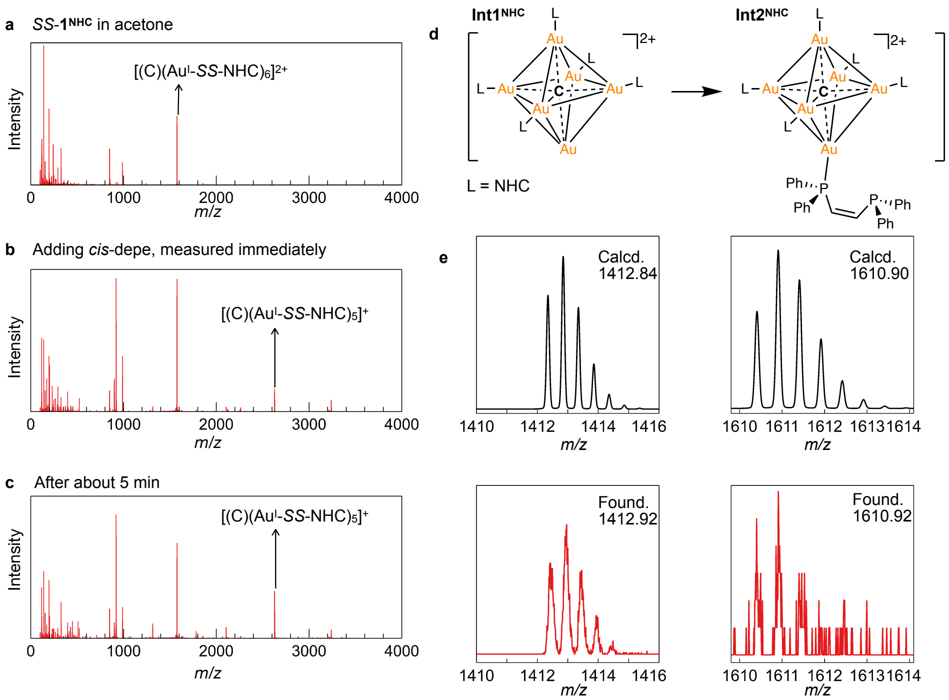

Supplementary Fig. 31. The ESI-MS spectra of monitoring the reaction of *SS*-**1**$^{\mathbf{NHC}}$ etched with *cis*-depe. **a-c** The ESI-MS results. **d** The proposed intermediates. **e** The simulated isotope patterns of **Int1**$^{\mathbf{NHC}}$ and **Int2**$^{\mathbf{NHC}}$ and the corresponding experimental results.

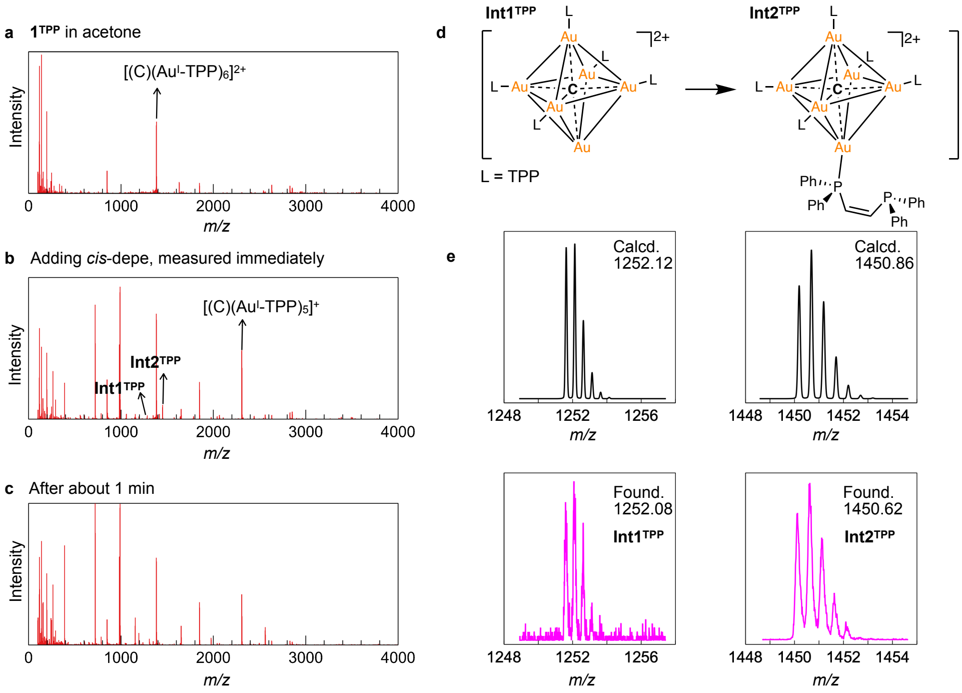

Supplementary Fig. 32. The ESI-MS spectra of monitoring the reaction of **1**$^{\mathbf{TPP}}$ and *cis*-depe. **a-c** The ESI-MS results. **d** The proposed intermediates. **e** The simulated isotope patterns of **Int1**$^{\mathbf{TPP}}$ and **Int2**$^{\mathbf{TPP}}$, and the corresponding experimental results.

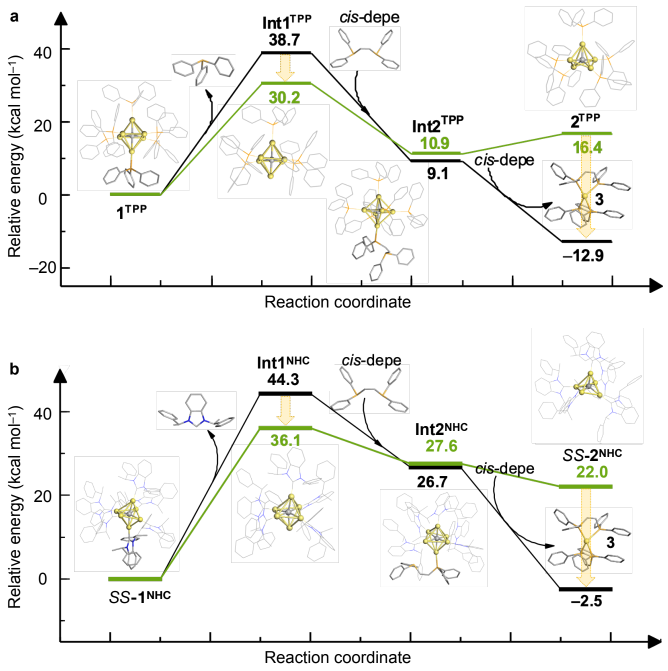

Supplementary Fig. 33. Calculated energy profiles for the proposed etching mechanism. **a** The energy profiles of etching $\mathbf{1}^{\mathbf{TPP}}$ with *cis*-depe (the green lines are relative energies in the solvent of the dichloromethane phase, and the black lines represent relative energies in the gas phase). **b** The energy profiles of etching *SS*-$\mathbf{1}^{\mathbf{NHC}}$ with *cis*-depe (the green lines are relative energies in the solvent of the dichloromethane phase, and the black lines represent relative energies in the gas phase). The above results indicate the different phases in dichloromethane and gas are critical in affecting the relative energies. The theoretical results are consistent with the experimental results that the $\mathbf{CAu^{I}_{5}}$ clusters (*SS*-$\mathbf{2}^{\mathbf{NHC}}$ and $\mathbf{2}^{\mathbf{TPP}}$) are very stable in the solid state, but less stable in dichloromethane. The spin states of all the involved species are the singlet state.

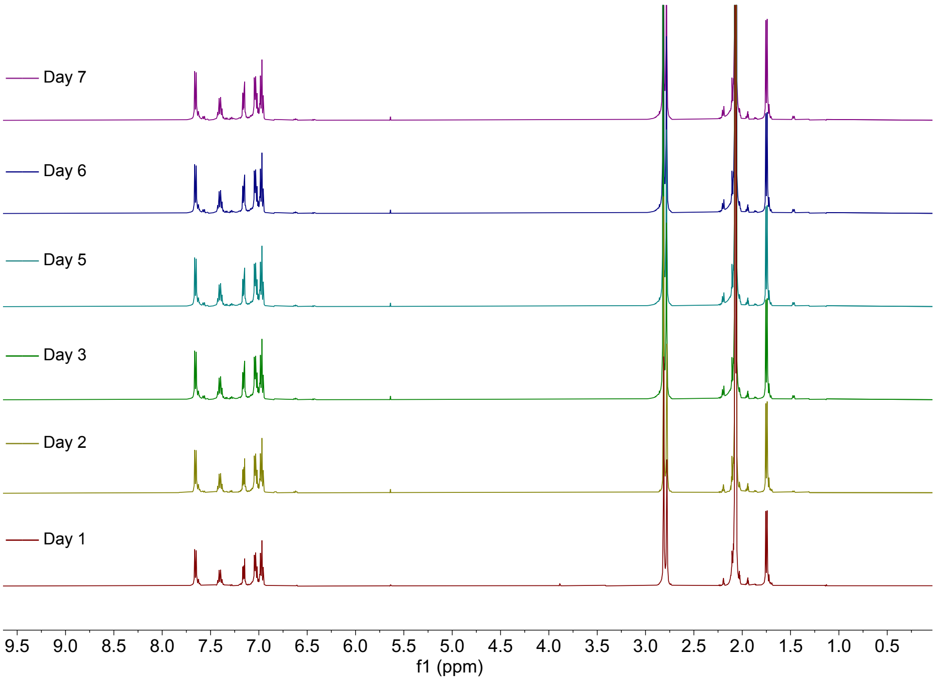

Supplementary Fig. 34. ^{1}H NMR spectra showing stability studies of *SS*-**2**$^{\mathbf{NHC}}$ in d_6-acetone for one week. Small signals (*) from *SS*-**1**$^{\mathbf{NHC}}$ can be observed.

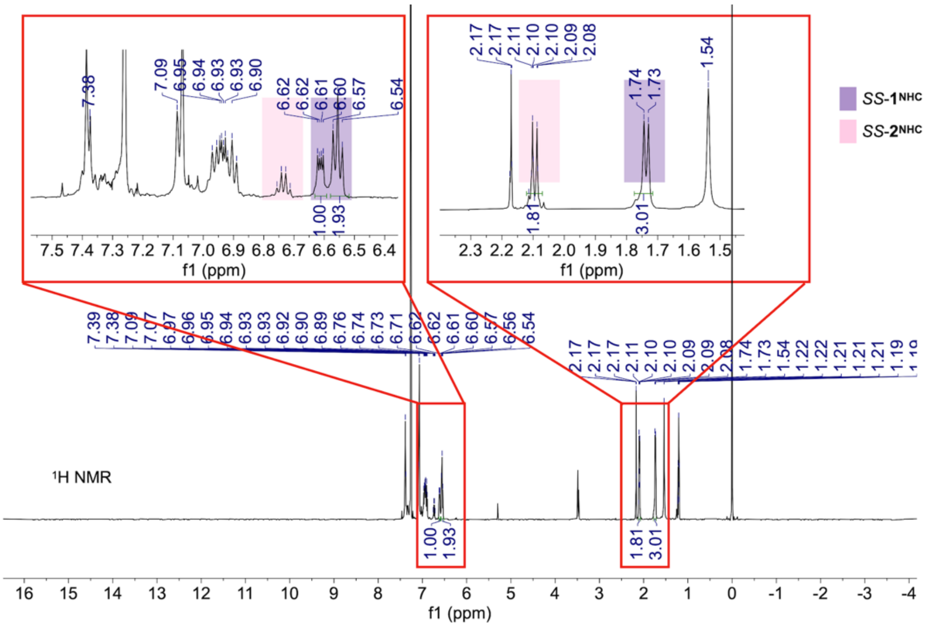

Supplementary Fig. 35. ^{1}H NMR spectrum of *SS*-**2**$^{\mathbf{NHC}}$ in $CDCl_3$ (500 M, 300 K). Once the crystal of *SS*-**2**$^{\mathbf{NHC}}$ was dissolved in $CDCl_3$, the spectrum was immediately measured. Based on the ratio of $–CH_3$ groups in *SS*-**1**$^{\mathbf{NHC}}$ and *SS*-**2**$^{\mathbf{NHC}}$, the ratio of *SS*-**1**$^{\mathbf{NHC}}$ to *SS*-**2**$^{\mathbf{NHC}}$ is calculated as 1.4.

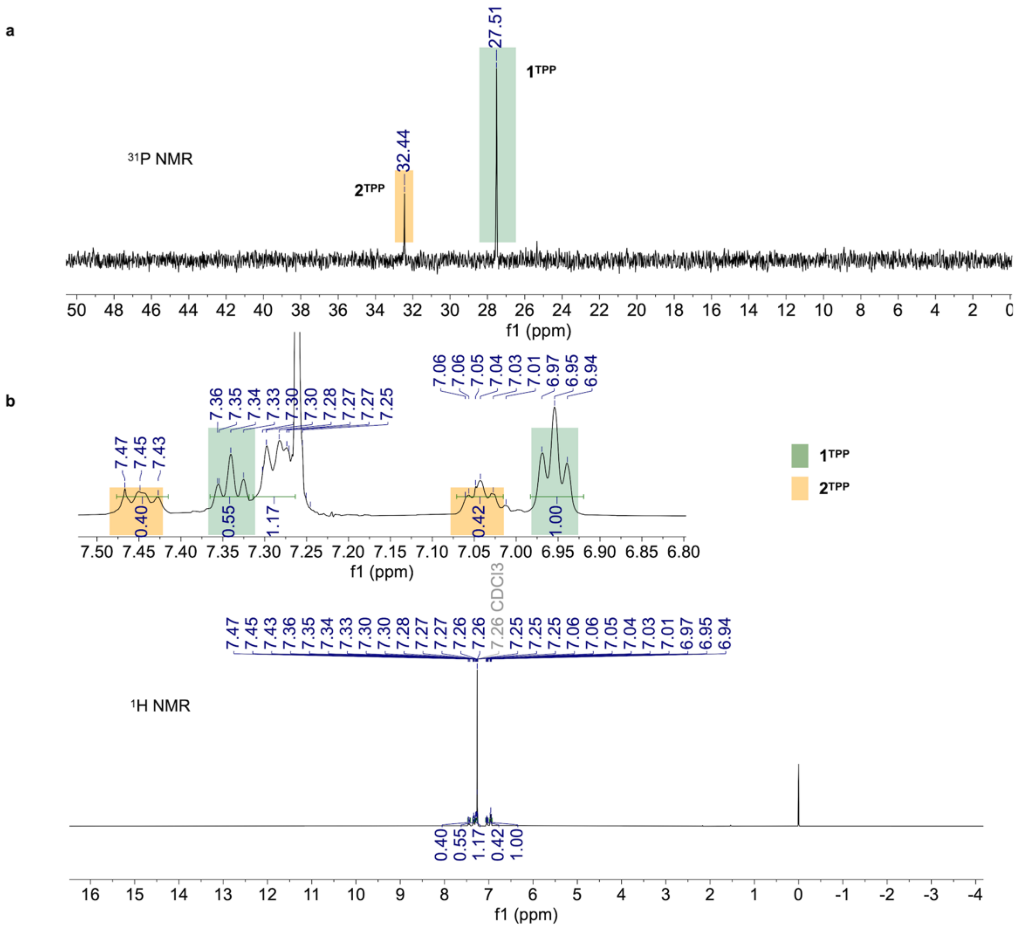

Supplementary Fig. 36. ^{31}P NMR and ^{1}H NMR spectra of crystalline **2**$^{\mathbf{TPP}}$ in $CDCl_3$ (300 K). Once the crystal of **2**$^{\mathbf{TPP}}$ was dissolved in $CDCl_3$, the spectra were immediately measured. **a** ^{31}P NMR spectrum (202 M, 300 K) showing signals from **1**$^{\mathbf{TPP}}$ and **2**$^{\mathbf{TPP}}$. **b** ^{1}H NMR spectrum (500 M, 300 K), based on the ratio of Ph groups in **1**$^{\mathbf{TPP}}$ and **2**$^{\mathbf{TPP}}$, the ratio of **1**$^{\mathbf{TPP}}$ to **2**$^{\mathbf{TPP}}$ is calculated as 2.1.

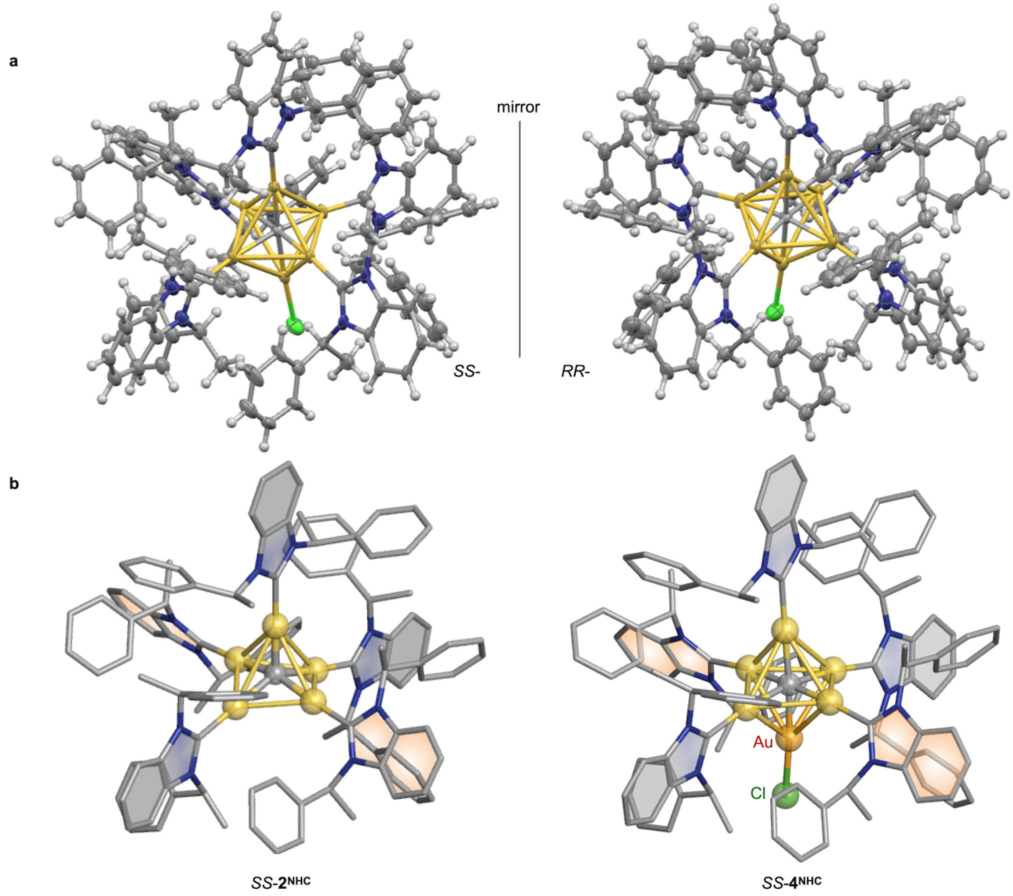

Supplementary Fig. 37. Single-crystal X-ray structures of the Cl-coordinated $\mathbf{CAu^{I}_{6}}$ clusters. **a** Cationic parts in *SS*-$\mathbf{4}^{\mathrm{NHC}}$ and *RR*-$\mathbf{4}^{\mathrm{NHC}}$ at the 50% thermal ellipsoid probability. **b** Comparison of cations between *SS*-$\mathbf{2}^{\mathrm{NHC}}$ and *SS*-$\mathbf{4}^{\mathrm{NHC}}$. Colour code: Au, yellow; C, grey; N, blue; P, orange; H, white; Cl, green. BF_4^- and solvent molecules are omitted for clarity.

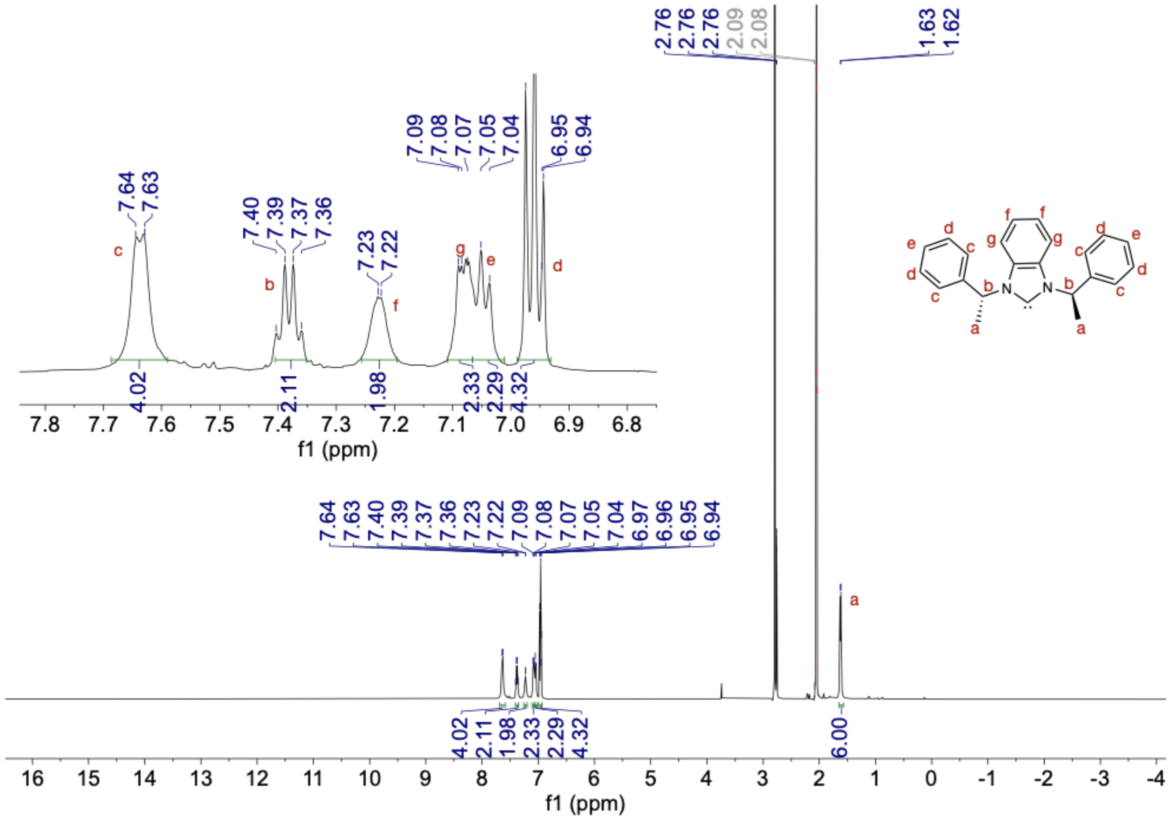

Supplementary Fig. 38. ^{1}H NMR spectrum of *SS*-$\mathbf{4}^{\mathbf{NHC}}$ in d_6-acetone (500 MHz, 300 K).

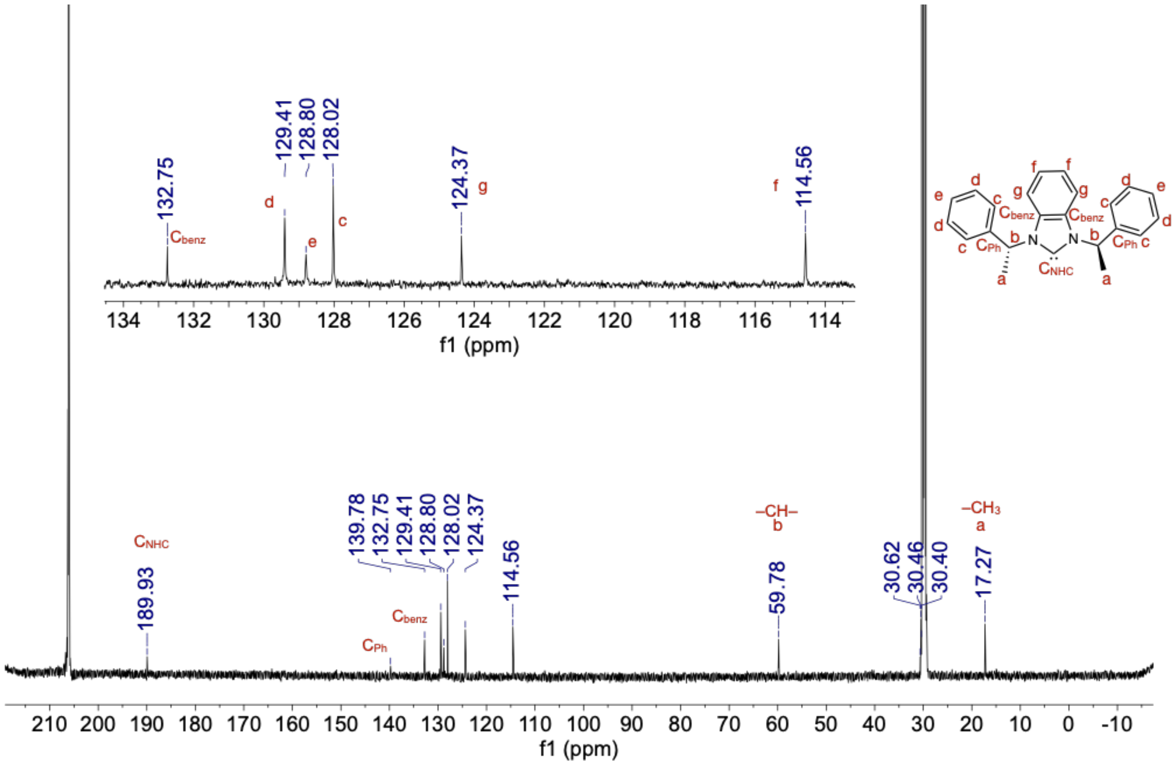

Supplementary Fig. 39. ^{13}C NMR spectrum of *SS*-$\mathbf{4}^{\mathbf{NHC}}$ in d_6-acetone (126 MHz, 300 K).

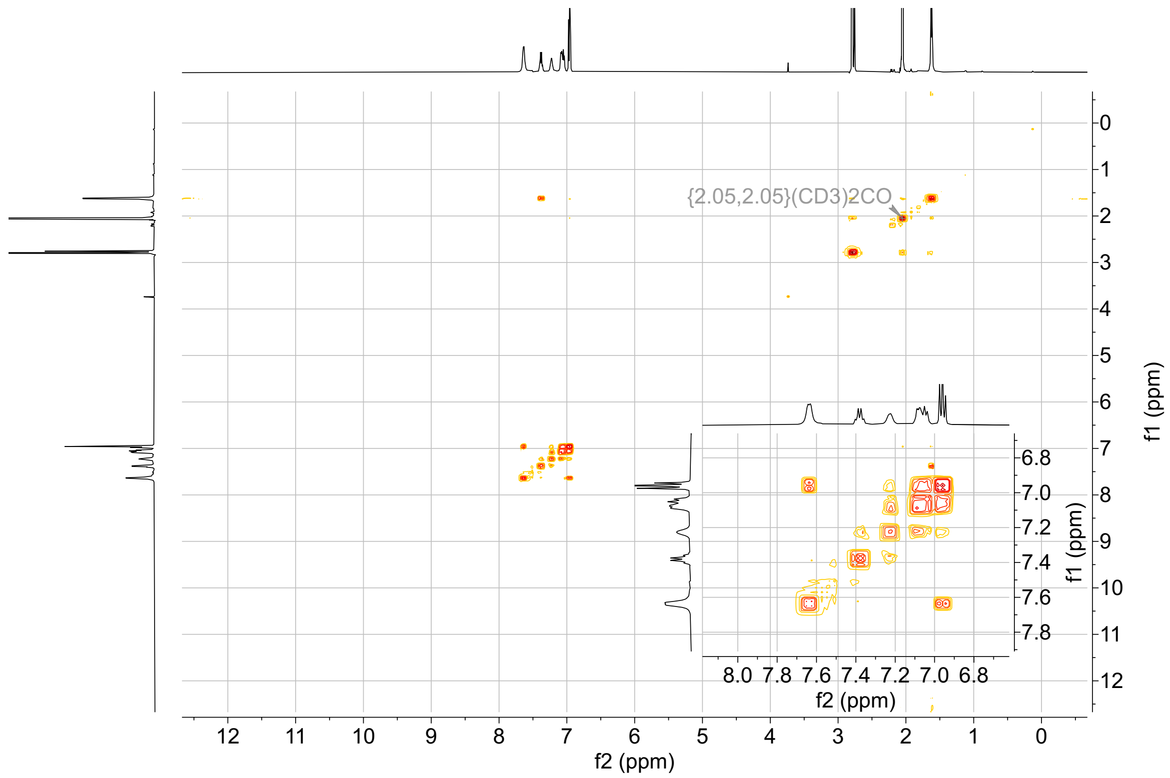

Supplementary Fig. 40. ^{1}H–^{1}H COSY NMR spectrum of *SS*-**4**$^{\mathbf{NHC}}$ in d_6-acetone (300 K).

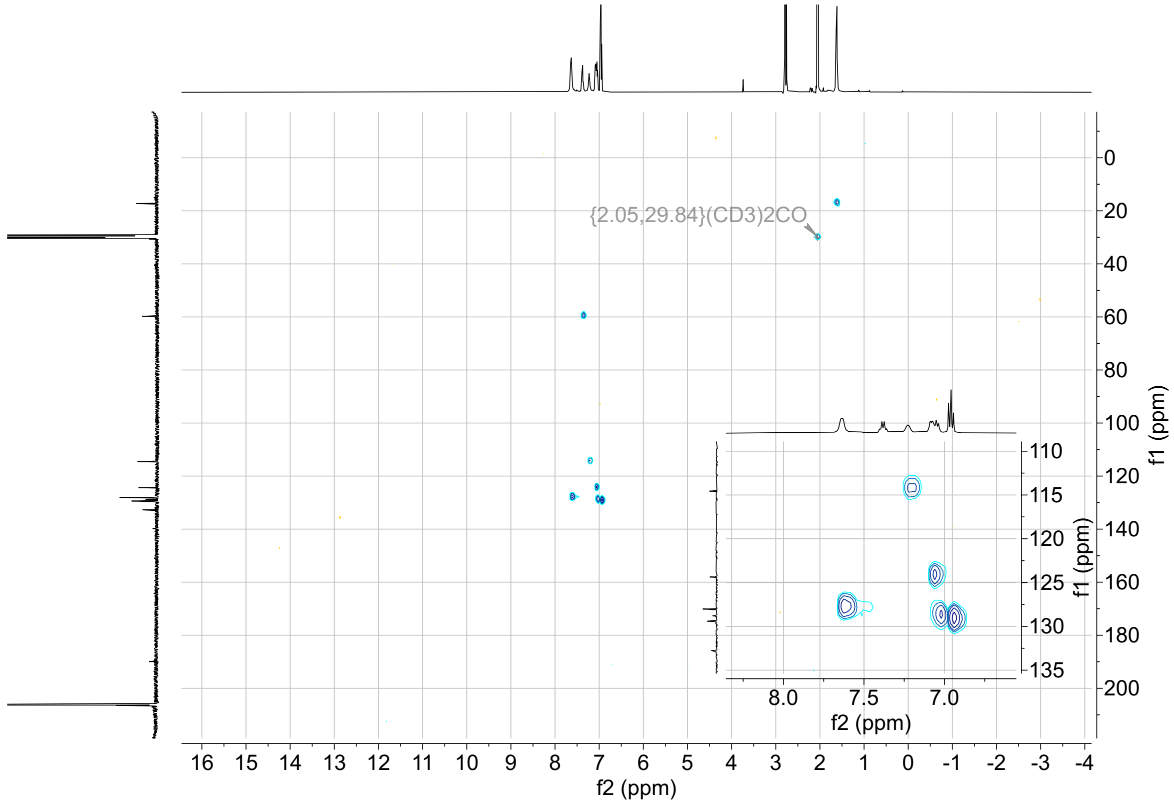

Supplementary Fig. 41. ^{13}C–^{1}H HSQC NMR spectrum of *SS*-**4**$^{\mathbf{NHC}}$ in d_6-acetone (300 K).

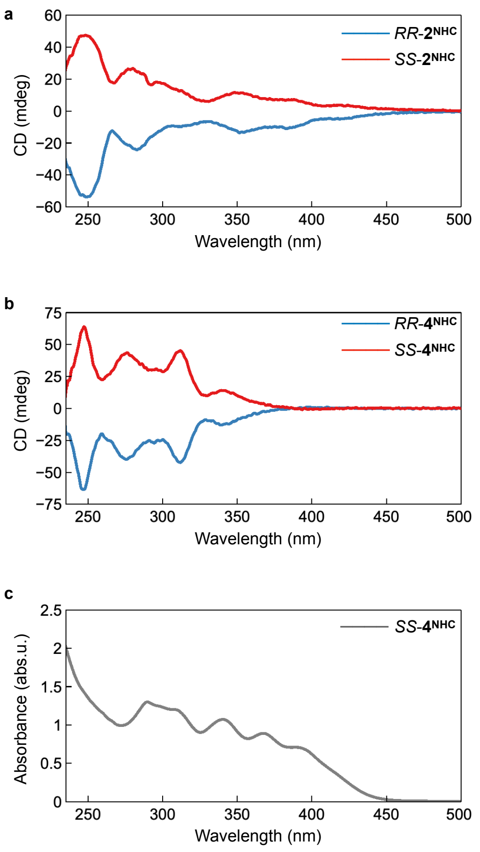

Supplementary Fig. 42. CD spectra of the *C*-centred gold(I) clusters. **a** *SS*- and *RR*-**2**$^{\mathbf{NHC}}$ in CH_2Cl_2 (c = 20 μM, 293 K). **b** *SS*- and *RR*-**4**$^{\mathbf{NHC}}$ in CH_2Cl_2. **c** The UV-vis absorption spectrum of *SS*-**4**$^{\mathbf{NHC}}$ in CH_2Cl_2 (c = 20 μM, 293 K).

Supplementary Tables

Supplementary Table 1. Crystallographic data of *SS*-**2**$^{\mathbf{NHC}}$, *RR*-**2**$^{\mathbf{NHC}}$ and **2**$^{\mathbf{TPP}}$.

Identification code	*SS*-**2**$^{\mathbf{NHC}}$	*RR*-**2**$^{\mathbf{NHC}}$	**2**$^{\mathbf{TPP}}$
Empirical formula	$C_{116}H_{110}Au_5BF_4N_{10}$·2.5($CH_2Cl_2$)·0.5($H_2O$) [a]	$C_{116}H_{110}Au_5BF_4N_{10}$·2.25($H_2O$) [b]	$C_{91}H_{75}Au_5BF_4P_5$
Formula weight	2937.19 [a]	2756.40 [b]	2395.00
Temperature (K)	93	93	93
Crystal system	tetragonal	tetragonal	monoclinic
Space group	*I*4	*I*4	$P2_1/n$
a (Å)	21.35370(10)	21.07880(10)	20.0525(5)
b (Å)	21.35370(10)	21.07880(10)	15.4181(5)
c (Å)	25.36310(10)	25.0288(3)	26.5756(7)
α (°)	90	90	90
β (°)	90	90	96.145(2)
γ (°)	90	90	90
Volume (Å^3)	11565.08(12)	11120.69(17)	8169.2(4)
Z	4	4	4
ρ_{calc} (g cm^{-3})	1.678	1.644	1.947
μ (mm^{-1})	13.152	12.569	9.100
F(000)	5648.0	5320.0	4528.0
Crystal size (mm^3)	0.1 × 0.1 × 0.1	0.1 × 0.1 × 0.1	0.2 × 0.2 × 0.2
Radiation	Cu*Kα* (λ = 1.54184)	Cu*Kα* (λ = 1.54184)	Mo*Kα* (λ = 0.71073)
2Θ range for data collection (°)	5.41 to 144.592	5.93 to 144.486	4.394 to 59.996
Index ranges	$-26 \leq h \leq 26$, $-26 \leq k \leq 26$, $-29 \leq l \leq 30$	$-25 \leq h \leq 26$, $-25 \leq k \leq 25$, $-30 \leq l \leq 26$	$-27 \leq h \leq 26$, $-16 \leq k \leq 20$, $-33 \leq l \leq 34$
Reflections collected	124691	57066	76684
Independent reflections	10850 [R_{int} = 0.0958, R_{sigma} = 0.0316]	9294 [R_{int} = 0.0559, R_{sigma} = 0.0293]	20683 [R_{int} = 0.0333, R_{sigma} = 0.0349]
Data/restraints/parameters	10850/457/602	9294/576/612	20683/0/775
Goodness-of-fit on F^2	1.089	1.045	1.022
Final *R* indexes [$I >= 2\sigma(I)$]	R_1 = 0.0915, wR_2 = 0.2310	R_1 = 0.0582, wR_2 = 0.1565	R_1 = 0.0230, wR_2 = 0.0425
Final *R* indexes [all data]	R_1 = 0.0931, wR_2 = 0.2340	R_1 = 0.0663, wR_2 = 0.1653	R_1 = 0.0339, wR_2 = 0.0447
Largest diff. peak/hole (e Å^{-3})	4.59/-2.75	2.65/-0.97	1.20/-0.93
Flack parameter	0.010(9)	-0.027(11)	
CCDC deposition number	2280948	2280949	2280950

Note: a, The hydrogen atoms in the dichloromethane and water solvent molecules were not located, but are included in the formula. b, The hydrogen atoms in the water solvent molecules were not located, but are included in the formula.

Supplementary Table 2. Important bond lengths and angles of *SS*-**2**^{NHC}, *RR*-**2**^{NHC} and **2**^{TPP}.

	SS-**2**^{NHC}	*RR*-**2**^{NHC}	**2**^{TPP}
$Au^I \cdots Au^I$ distances (Å)	2.8667(10) – 3.3141(15)	2.8927(10) – 3.3321(15)	2.85528(18) – 3.21332(19)
C_{centre}–Au^I (Å)	2.03(2) – 2.075(8)	2.01(2) – 2.089(8)	2.064(3) – 2.082(3)
C_{NHC}–Au^I (Å)	2.03(2) – 2.08(4)	2.03(4) – 2.077(19)	–
P–Au^I (Å)	–	–	2.2546(8) – 2.2735(8)
Au^I–C_{centre}–Au^I (°)	87.4(3) – 163.7(14)	88.2(2) – 172.3(13)	86.95(10) – 174.31(16)

Supplementary Table 3. Bond length (d, Å) and Wiberg bond order (WBO) of C_{centre}–Au^I and $Au^I \cdots Au^I$ in different CAu^I_n (n = 5, 6) clusters.

	I*i*Pr–**CAu$^I_6$**[5]		*SS*-**1**^{NHC}		*SS*-**2**^{NHC}	
	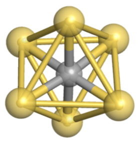		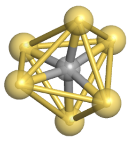		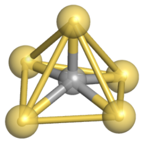	
	d	WBO	d	WBO	d	WBO
C_{centre}–Au^I	2.19	0.41	2.22	0.40	2.09-2.16	0.50-0.57
$Au^I \cdots Au^I$	3.10	0.16	2.95-3.45	0.12-0.18 (avg. 0.16)	2.96-3.48	0.13-0.20 (avg. 0.18)
			**1**^{TPP}		**2**^{TPP}	
			d	WBO	d	WBO
C_{centre}–Au^I			2.19	0.40	2.10-2.16	0.50-0.55
$Au^I \cdots Au^I$			3.06-3.13	0.15	3.00-3.65	0.10-0.18 (avg. 0.16)

Note: Wiberg bond orders were obtained by NBO 3.1[6] as implemented in Gaussian 16.

Supplementary Table 4. Excited states of *SS*-**2**$^{\mathbf{NHC}}$ with oscillator strength (*f*) larger than 0.04 calculated by TD-DFT.

State number	*λ* (nm)	Δ*E* (eV)	*f*	Transition character
2	415	2.988	0.1780	H−1→L
3	402	3.084	0.2933	H−2→L
4	393	3.152	0.2647	H→L+1
6	363	3.420	0.2269	H−2→L+1
11	333	3.727	0.1735	H−1→L+3
13	329	3.771	0.0524	H−2→L+2
14	325	3.810	0.1335	H−2→L+3
15	324	3.825	0.1634	H→L+5
17	313	3.961	0.0787	H→L+7
20	307	4.035	0.0530	H−1→L+5
75	272	4.552	0.1748	H−5→L
76	272	4.554	0.0452	H−2→L+19
84	269	4.617	0.040	H→L+32
94	266	4.668	0.2829	H−12→L
96	265	4.676	0.0543	H−10→L
97	264	4.695	0.1277	H−11→L
102	260	4.760	0.0774	H−3→L+4
103	260	4.766	0.0634	H−4→L+1
105	259	4.795	0.1234	H−5→L+1
137	247	5.015	0.1237	H−11→L+1
155	243	5.109	0.0768	H−4→L+4
177	236	5.249	0.0516	H−24→L+1, H−6→L+4

Supplementary Table 5. Excited states of $\mathbf{2}^{\mathrm{TPP}}$ with oscillator strength (f) larger than 0.04 calculated by TD-DFT.

State number	λ (nm)	ΔE (eV)	f	Transition character
4	347	3.569	0.1698	H−1→L, H→L+1
32	303	4.088	0.1118	H→L+13
33	302	4.103	0.0543	H−2→L+9, H−1→L+11
34	302	4.106	0.0666	H−1→L+11, H−2→L+9
41	296	4.187	0.2400	H−2→L+10
46	288	4.306	0.1047	H−2→L+11
48	287	4.319	0.0545	H→L+17
57	281	4.409	0.0677	H−1→L+19
59	281	4.415	0.1198	H−1→L+20
60	281	4.417	0.1018	H−1→L+18
89	261	4.748	0.0684	H−3→L+2, H−1→L+31
90	261	4.751	0.0444	H−1→L+31
92	261	4.756	0.0964	H−3→L+3
95	259	4.781	0.0517	H−3→L+4
105	257	4.832	0.2554	H−3→L+9
106	255	4.853	0.0494	H→L+32, H→L+33
107	255	4.857	0.0606	H−1→L+32
112	252	4.929	0.0459	H−2→L+30
113	251	4.932	0.0902	H−4→L+1
119	246	5.033	0.0689	H−6→L
122	246	5.044	0.0786	H−6→L+1
127	243	5.097	0.0829	H−5→L+4, H−4→L+7
128	243	5.099	0.0427	H−5→L+7
190	227	5.452	0.0902	H→L+35
191	227	5.453	0.0456	H→L+35
193	227	5.464	0.0464	H−1→L+35
195	227	5.470	0.0549	H−1→L+35

Supplementary Table 6. Orbital composition analysis with Mulliken partition of *SS*-**2**^{NHC}.

	Au	carbon-centre	NHC
LUMO+3	6.8%	0.2%	93.0%
LUMO+2	7.2%	0.8%	92.0%
LUMO+1	25.0%	0.1%	74.8%
LUMO	35.2%	0.1%	64.7%
HOMO	55.4%	32.5%	12.1%
HOMO–1	53.0%	29.7%	17.4%
HOMO–2	52.6%	26.9%	20.6%
HOMO–3	79.9%	0.1%	19.9%

Supplementary Table 7. Orbital composition analysis with Mulliken partition of **2**^{TPP}.

	Au	carbon-centre	TPP
LUMO+3	1.1%	0.1%	98.7%
LUMO+2	1.5%	0.1%	98.4%
LUMO+1	22.4%	0.0%	77.5%
LUMO	22.8%	0.0%	77.2%
HOMO	51.0%	32.3%	16.7%
HOMO–1	50.6%	32.2%	17.1%
HOMO–2	51.4%	30.1%	18.6%
HOMO–3	53.4%	0.1%	46.6%

Supplementary Table 8. Orbital composition analysis with Mulliken partition of **1**^{TPP}.

	Au	carbon-centre	TPP
LUMO+3	2.9%	0.1%	97.1%
LUMO+2	43.0%	0.1%	56.9%
LUMO+1	43.3%	0.1%	56.6%
LUMO	42.5%	0.1%	57.4%
HOMO	46.7%	29.2%	24.0%
HOMO–1	46.5%	29.6%	24.0%
HOMO–2	48.6%	29.4%	22.0%
HOMO–3	42.3%	0.2%	57.5%

Supplementary Table 9. TD-DFT calculated emission values of *SS*-**1**^{NHC} and *SS*-**2**^{NHC}.

	Calculated		Experimental	
	nm	eV	nm	eV
SS-**1**^{NHC}	609	2.04	525	2.36
SS-**2**^{NHC}	691	1.79	676	1.83

Supplementary Table 10. Crystallographic data of *SS*-**4**$^{\mathbf{NHC}}$ and *RR*-**4**$^{\mathbf{NHC}}$.

Identification code	*SS*-**4**$^{\mathbf{NHC}}$	*RR*-**4**$^{\mathbf{NHC}}$
Empirical formula	$C_{116}H_{110}Au_6BClF_4N_{10}\cdot 4(CH_3COCH_3)$	$C_{116}H_{110}Au_6BClF_4N_{10}\cdot 4(CH_3COCH_3)$
Formula weight	3180.60	3181.60
Temperature (K)	93	93
Crystal system	monoclinic	monoclinic
Space group	$P2_1$	$P2_1$
a (Å)	16.3948(2)	16.4675(3)
b (Å)	17.01066(19)	17.0294(2)
c (Å)	21.6028(2)	21.6245(2)
α (°)	90	90
β (°)	95.2435(12)	95.4511(13)
γ (°)	90	90
Volume (Å^3)	5999.52(12)	6036.78(14)
Z	2	2
ρ_{calc} (g cm^{-3})	1.761	1.750
μ (mm^{-1})	14.142	14.054
F(000)	3072.0	3072.0
Crystal size (mm^3)	0.01 × 0.01 × 0.01	0.01 × 0.01 × 0.01
Radiation	Cu*Kα* (*λ* = 1.54184)	Cu*Kα* (*λ* = 1.54184)
2*Θ* range for data collection (°)	5.414 to 144.918	5.39 to 144.51
Index ranges	$-20 \le h \le 19$, $-20 \le k \le 20$, $-26 \le l \le 24$	$-19 \le h \le 19$, $-21 \le k \le 20$, $-26 \le l \le 20$
Reflections collected	57723	48169
Independent reflections	22817 [R_{int} = 0.0408, R_{sigma} = 0.0443]	19780 [R_{int} = 0.0576, R_{sigma} = 0.0574]
Data/restraints/parameters	22817/731/1424	19780/733/1404
Goodness-of-fit on F^2	1.062	1.014
Final *R* indexes [$I >= 2\sigma(I)$]	R_1 = 0.0756, wR_2 = 0.1943	R_1 = 0.0873, wR_2 = 0.2169
Final *R* indexes [all data]	R_1 = 0.0787, wR_2 = 0.1984	R_1 = 0.0916, wR_2 = 0.2226
Largest diff. peak/hole（e Å^{-3})	4.53/-2.61	5.47/-2.65
Flack parameter	0.013(19)	0.02(2)
CCDC deposition number	2280951	2280952

Supplementary Table 11. Important bond lengths and angles of *SS*-**4**$^{\mathbf{NHC}}$ and *RR*-**4**$^{\mathbf{NHC}}$.

	SS-**4**$^{\mathbf{NHC}}$	*RR*-**4**$^{\mathbf{NHC}}$
$Au^{I}\cdots Au^{I}$ distances (Å)	2.8738(11) – 3.1436(11)	2.8732(12) – 3.1435(12)
C_{centre}–Au^{I} (Å)	2.05(2) – 2.159(18)	2.05(2) – 2.16(2)
C_{NHC}–Au^{I} (Å)	2.01(3) – 2.067(18)	2.01(2) – 2.11(2)
Au^{I}–Cl (Å)	2.308(6)	2.310(7)
Au^{I}–C_{centre}–Au^{I} (°)	87.9(9) – 175.9(10)	84.7(6) – 175.3(10)

Supplementary Note

SS-**2**$^{\mathbf{NHC}}$

PLAT306_ALERT_2_B Isolated Oxygen Atom (H-atoms Missing ?) .... O11 Check

Author Response: This alert is because of the solvent water molecule.

PLAT342_ALERT_3_B Low Bond Precision on C-C Bonds .... 0.02425 Ang.

Author Response: This alert is due to the limited diffraction resolution, particularly the weak diffraction at the high angle of the determined crystals.

RR-**4**$^{\mathbf{NHC}}$

PLAT306_ALERT_2_B Isolated Oxygen Atom (H-atoms Missing ?) .... O3 Check

Author Response: This alert is because of the solvent water molecule.

PLAT342_ALERT_3_B Low Bond Precision on C-C Bonds .... 0.02588 Ang.

Author Response: This alert is due to the limited diffraction resolution, particularly the weak diffraction at the high angle of the determined crystals.

SS-**4**$^{\mathbf{NHC}}$

PLAT342_ALERT_3_B Low Bond Precision on C-C Bonds .... 0.03595 Ang.

Author Response: This alert is due to the limited diffraction resolution, particularly the weak diffraction at the high angle of the determined crystals.

RR-**4**$^{\mathbf{NHC}}$

PLAT342_ALERT_3_B Low Bond Precision on C-C Bonds .... 0.04241 Ang.

Author Response: This alert is due to the limited diffraction resolution, particularly the weak diffraction at the high angle of the determined crystals.

Supplementary References

1. Pei, X.-L. et al. Asymmetric twisting of *C*-centered octahedral gold(I) clusters by chiral *N*-heterocyclic carbene ligation. *J. Am. Chem. Soc.* **144**, 2156–2163 (2022).
2. Scherbaum, F., Grohmann, A., Huber, B., Krüger, C. & Schmidbaur, H. “Aurophilicity” as a consequence of relativistic effects: the hexakis(triphenylphosphaneaurio) methane dication $[(Ph_3PAu)_6C]^{2\oplus}$. *Angew. Chem. Int. Ed. Engl.* **27**, 1544–1546 (1988).
3. Scherbaum, F., Grohmann, A., Müller, G. & Schmidbaur, H. Synthesis, structure, and bonding of the cation $[\{(C_6H_5)_3PAu\}_5C]^{\oplus}$. *Angew. Chem. Int. Ed. Engl.* **28**, 463–465 (1989).

4. Healy, P. C., Bradley T., Michael L. W. & Peter G. P. Synthesis, structure and cytotoxicity studies of four-coordinate bis(cis–bis(diphenylphosphino)ethene) gold(I) complexes. $[Au(dppey)_2]X$. *J. Inorg. Biochem.* **104**, 625–631 (2010).

5. Ube, H., Zhang, Q. & Shionoya, M. A carbon-centered hexagold(I) cluster supported by *N*-heterocyclic carbene ligands. *Organometallics* **37**, 2007–2009 (2018).

6. Glendening, E. D., Reed, A. E., Carpenter, J. E. & Weinhold, F. NBO Version 3.1 (2001).
